# Integrating morphological and phytochemical characters in understanding the taxonomic relationship within some *Ipomoea* L. species

**DOI:** 10.1186/s12870-026-08857-4

**Published:** 2026-05-05

**Authors:** Mohamed A. Salim, Mona O. El Shabrawy, Salma S. Abd El-Ghany, Mariam I. Hussein, Mona M. Marzouk

**Affiliations:** 1 Department of Botany, Faculty of Science, Ain Shams University, Cairo, Egypt; 2 Department of Phytochemistry and Plant Systematics, National Research Centre, P.O. 12622, 33 El Bohouth St, Cairo, Egypt

**Keywords:** Flavonoids, *Ipomoea*, Lamina, Micromorphology, Stem

## Abstract

**Background:**

*Ipomoea* is a large and diverse genus within the family Convolvulaceae, widely utilized for ornamental, nutritional, and medicinal purposes. The genus exhibits considerable variation in stem and lamina structures, reflecting its ecological diversity and growth habits. This variability has contributed to taxonomic uncertainty at the infrageneric level. The present study aims to identify diagnostic morphological characters of the stem and lamina in eight Egyptian wild and cultivated *Ipomoea* species, and to evaluate their secondary metabolite profiles to assess their taxonomic significance. Macro- and micromorphological characters were examined using light and scanning electron microscopy. Phytochemical profiling was conducted using liquid chromatography–electrospray ionization tandem mass spectrometry (LC-ESI-MS/MS), supported by molecular networking analysis.

**Result:**

Several macro- and micromorphological characters were diagnostic at the species level, including plant habit, leaf composition, lamina shape, nectary gland, trichome type, vascular system, pith type, and secretory cell distribution. Furthermore, LC-ESI-MS/MS supported by molecular networking led to the annotation of 132 metabolites, of which 107 were secondary metabolites, substantially expanding the known chemical profiles of the studied species. Notably, this study provides the first comprehensive chemical description of *Ipomoea ochracea*. Finally, integration of morphological and phytochemical data into a dendrogram revealed a coherent hierarchical structure reflecting varying degrees of affinity among the species. Remarkably, *I. tricolor* was distinctly separated from the remaining species, which formed a large cluster further subdivided into two major groups.

**Conclusion:**

These findings demonstrate that the combined characters provide reliable and complementary criteria for species delimitation within *Ipomoea*, highlighting the significance of integrative approaches in plant systematics. Further studies incorporating additional datasets, particularly molecular analyses based on chloroplast and nuclear DNA markers, are recommended to achieve a more comprehensive understanding of relationships within the genus.

**Supplementary Information:**

The online version contains supplementary material available at 10.1186/s12870-026-08857-4.

## Background

Convolvulaceae Juss., the bindweed or morning glory family, is a large cosmopolitan family comprising about 60 genera and approximately 2000 species [1, 2]. Most genera are weeds, creeping vines or climbers, with white latex, simple alternate leaves, and showy flowers with infundibuliform corollas [1]. The presence of articulated latex canals or latex cells is considered a unique feature in Convolvulaceae [3].

*Ipomoea* L. is one of the largest and most diverse genera within the family, with 600–800 species [2, 4]. The genus *Ipomoea* is widely used for ornamental, nutritional, and medicinal purposes, such as a laxative [5, 6]. Additionally, the latex of some *Ipomoea* species is utilized in the vulcanization of rubber [7]. One of the most economically significant *Ipomoea* species is *I. batatas* (L.) Lam. (the sweet potato), which is cultivated globally for its edible storage roots and serves as a staple crop in many tropical and subtropical regions [6]. Other well-known species, such as *I. purpurea* and *I. tricolor*, are valued ornamental plants for their attractive, colorful flowers [8]. Morphologically, species of *Ipomoea* are herbaceous, lianescent and climbing plants, and a small group of shrubs and trees. It is characterized by simple alternate leaves and large, showy, funnel-shaped flowers, typically with a sympetalous corolla, five epipetalous stamens, and a superior ovary with two carpels and a permanent calyx [9]. Anatomical investigations of *Ipomoea* species have revealed a variety of structural adaptations in the stem and lamina, reflecting the ecological diversity and growth habits of these plants. Generally, *Ipomoea* species have a typical eustelic vascular structure, with open collateral vascular bundles arranged in a ring [10]. The presence of bicollateral vascular bundles has also been reported in some species, such as *I. cairica*, indicating specialized vascular differentiation [11]. Commonly, in Convolvulaceae, the intra-xylary phloem (internal phloem**)** at the margin of the pith is well documented and considered a specific character of the family. During the secondary growth, some taxa develop an internal cambium found below the protoxylem that divides unidirectionally (phloem only) or bidirectionally (xylem outward, phloem inward), supporting climbing or arborescent habits [12–14]. The lamina in *Ipomoea* species exhibits a typical dorsiventral leaf structure, with palisade parenchyma adaxially and spongy parenchyma abaxially [15]. Sun-exposed species such as *I. carnea* show additional xeromorphic adaptations, including a thickened cuticle, sunken stomata, compact mesophyll that reflects habitat-driven plasticity, and vascular bundles enclosed in a fibrous sheath, which contributes to structural rigidity [16]. Laticifers are consistently observed in the cortex, phloem, and pith of stems [10]. Although latex production is linked to defense and wound response, for example, *I. carnea* produces a white or yellowish latex documented to contain bioactive compounds. Their production may support species delimitation when combined with other characters [17, 18].

Epidermal studies can provide additional information in ascertaining the systematic position of disputed taxa [19]. Few studies have focused on the epidermal features of the *Ipomoea* lamina, while many studies are largely limited to stomatal ontogeny. Stomatal form and shape, associated epidermal cells, guard cell morphology, stomatal frequency, and trichome type and distribution are valuable taxonomic tools [20, 21]. Stomata are typically located on both ad- and abaxial leaf surfaces, with a higher frequency abaxially. They are usually paracytic, diacytic, anisocytic, and occasionally anomocytic, depending on the species [22]. Moreover, trichomes are frequent on both surfaces of the lamina, varying in type, density, and distribution. These structures contribute not only to species identification but also to ecological functions such as protection against herbivory and drought [23].

From a phytochemical point of view, *Ipomoea* species are rich sources of diverse natural products, including saponins, flavonoids, phenolic acids, and alkaloids [24, 25]. Consequently, they have demonstrated significant pharmacological activities [26–30], highlighting their potential dietary and medicinal value.

LC-ESI-MS/MS is a powerful analytical technique widely used across various scientific disciplines. It combines liquid chromatography with electrospray ionization and tandem mass spectrometry, offering high sensitivity and flexibility in the analysis of complex mixtures. This technique has diverse applications, including pharmaceutical analysis, environmental studies, metabolomics, and proteomics [31]. Moreover, the molecular networking (MN) strategy implemented on the GNPS (global natural product social molecular networking) platform represents an advanced bioinformatic approach that organizes tandem mass spectrometry data based on chemical similarity [32, 33]. It facilitates the rapid identification of known compounds and their analogues, addressing major challenges for compounds visualization and annotation in plant extracts [34, 35]. In addition, MN-based LC-ESI-MS/MS analysis has significantly advanced plant taxonomy by providing fast and reliable metabolite profiling tools for species discrimination, identification, and classification [36]. Nevertheless, the metabolic diversity of *Ipomoea* species remains insufficiently explored, and comprehensive phytochemical characterization of the genus is still lacking.

The integration of multiple datasets has become a fundamental approach in modern plant systematics, particularly in taxonomically complex groups. Although traditional morphological and anatomical characters remain essential for species identification, their diagnostic value may be limited by phenotypic plasticity and environmental influences [37]. In contrast, phytochemical traits provide relatively stable, genetically controlled markers that complement structural observations [38]. Despite these advances, most previous studies on *Ipomoea* species have relied on either morphological or phytochemical data independently, with limited efforts to integrate both datasets within a unified analytical framework. This lack of integrative approaches has contributed to persistent ambiguity in species delimitation. Dendrogram-based cluster analysis has proven to be an effective tool for synthesizing diverse datasets into a hierarchical framework, enabling visualization of relationships and identification of natural groupings among species [39]. Recent integrative analyses in genera such as *Ficus* L [40]., *Hibiscus* L [38]., *Reynoutria* Houtt [41]., and *Tanacetum* L [42]. have demonstrated that combining morphological and phytochemical datasets significantly improves species delimitation and reveals clearer patterns of affinity among taxa.

Molecular phylogenetic studies have further highlighted the taxonomic complexity of *Ipomoea*, demonstrating that the genus is not monophyletic [43–45]. Additionally, none of the three subgenera (*Eriospermum*, *Ipomoea*, and *Quamoclit*) as circumscribed by Austin and Huáman [46] has been supported as monophyletic [8, 47]. Instead, the genus *Ipomoea* has been reorganized into 18 sections, with higher subgeneric ranks failing to represent natural groups within the genus [4]. These findings underscore the need for complementary approaches to refine species boundaries and better understand relationships within the genus.

In this context, the present study addresses a clear research gap by integrating macro- and micromorphological characters with metabolomic profiling based on LC-ESI-MS/MS supported by molecular networking. Specifically, this study aims to identify diagnostic morphological characters of the stem and lamina of the studied *Ipomoea* species using LM and SEM, while simultaneously characterizing secondary metabolite profiles. Furthermore, dendrogram-based cluster analysis is employed to integrate these datasets into a unified framework, enabling the assessment of species affinities and the evaluation of their taxonomic significance. This integrative approach provides a more robust basis for species delimitation and contributes to a deeper understanding of structural and chemical diversity within *Ipomoea* species.

## Materials and methods

### Plant material

The fresh plant materials of eight wild and cultivated species were collected and identified by our research group. The wild species were identified according to Täckholm [48] and Boulos [49], while the identification of the cultivated species was done by Bailey [50] and Bailey & Bailey [51]. The voucher specimens of the collected species were deposited in the Herbarium of the National Research Centre (CAIRC). The synonyms, localities, date of collection, and voucher numbers of the studied species are shown in Table 1.

**Table 1:** Plant material and collection data of the studied species

No.	Taxa	Locality	Date of collection	Voucher No.
a	***Ipomoea batatas*** (L.) Lam. *=Batatas batatas* (L.) H.Karst. *=Convolvulus batatas* L.	Cultivated land, Kafr El-Sheikh governorate, Egypt	December 2024	M299
b *	***Ipomoea cairica*** (L.) Sweet =*Convolvulus cairicus* L. =*Exocroa* egyptiaca Raf.	Around irrigation canals, Kafr El-Sheikh governorate, Egypt	February 2024	1128
c *	***Ipomoea carnea*** Jacq. *=Convolvulus carneus* (Jacq.) Spreng.	Around irrigation canals, Kafr El-Sheikh governorate, Egypt	August 2024	1129
d *	***Ipomoea eriocarpa*** R.Br. *=Convolvulus eriocarpus* (R.Br.) Spreng.	Climber on *Zea mays* plant, Kafr El-Sheikh governorate, Egypt	December 2024	1129
e *	***Ipomoea imperati*** (Vahl) Griseb. *=Convolvulus imperati* Vahl *=Latrienda imperati* (Vahl) Raf. *=Ipomoea stolonifera* J.F.Gmel.	Sand dunes of coastal areas, Kafr El-Sheikh governorate, Egypt	August, 2024	1130
f	***Ipomoea ochracea*** (Lindl.) Sweet *=Convolvulus ochraceus* Lindl. *=Ipomoea curtisii* House	Mazhar botanic garden, Giza, Egypt	April 2024	M300
g	***Ipomoea pes-caprae*** (L.) R.Br. *=Convolvulus pes-caprae* L. *=Ipomoea aegopoda* St.Lag.	Ornamental pant nurseries, Giza, Egypt	March 2024	M301
h	***Ipomoea tricolor*** Cav. *=Convolvulus venustus* Spreng. =*Pharbitis tricolor* (Cav.) Chitt.	Orman botanic garden, Giza, Egypt	March, 2024	M302

(*) refers to wild taxa

### Morphological investigation

#### Macromorphological investigation

**Fig. 1:**
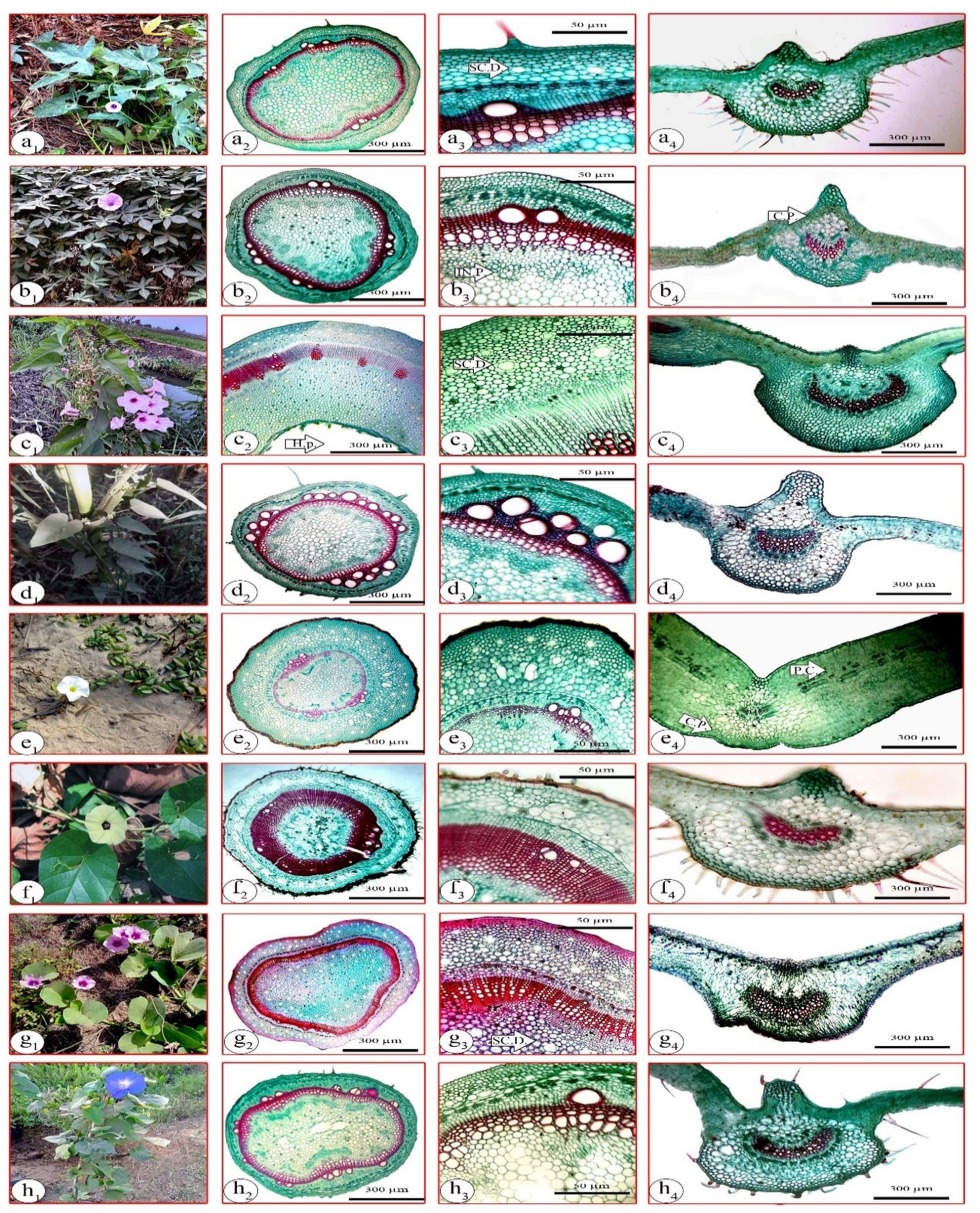
Macro- and microphotographs showing growth habits and different growth aspects of the stem and lamina of the studied species. **a**: *Ipomoea batatas*, **b**: *I. cairica*, **c**: *I. carnea*, **d**: *I. eriocarpa*, **e**: *I. imperati*, **f**: *I. ochracea*, **g**: *I. pes-caprae*, **h**: *I. tricolor*, 1: micromorphology, 2: T.S. of the stem (X = 300 μm), 3: detailed T.S. of the stem (X = 50 μm), 4: V.S. of the lamina (X = 300 μm). SC.D.: Schizogenous duct, C.P.: Continuous palisade cells, H.P.: Hollow pith, IN.P.: Included phloem, P.C.: Palisade cells

Macromorphological characters (Table 2) were carefully examined using fresh material, with at least five individuals per species evaluated to ensure representative observations. The collected species were photographed in their natural habitats using a Canon PowerShot A720, 8.0 megapixels (Fig. 1).

#### Micromorphological investigation

For anatomical characters, thin sections from the stem (between the fourth and fifth node), and lamina (at the middle portion) of the studied species were done by hand microtome (10–20 μm thick). The sections were double-stained using safranine and light green combination, then mounted with Canada Balsam [52]. Sections were examined and photographed using an Olympus C.35AD-2 microscope. The terminology of the internal structures followed Metcalfe and Chalk [9] and Koch et al. [53]. This work was done at the Botany Department, Faculty of Science, Ain Shams University.

For the lamina epidermis, strips were taken from the middle portion of the fresh lamina of the studied species, and the epidermal strips were prepared by mechanical stripping [52]. The epidermal strips were examined directly or fixed in FAA solution and stored in 70% ethanol until use, stained with (1%) safranine, then mounted on slides. The examinations and photomicrographs were taken using a Canon PowerShot A720, 8.0 megapixels. For SEM investigation, small pieces (7 mm^2^) of fresh lamina were excised from fully mature leaves and rinsed with ethanol to eliminate surface debris, then mounted on aluminum stubs with double-sided tape and coated with gold for 20 min with a film thickness of 5–10 nm in the SPI-Module sputter coater. The ab- and adaxial surfaces were observed and photographed by Scanning Electron Microscope (JSM-5500 LV; JEOL Ltd-Japan) by using high vacuum mode. Terminology of the epidermal characteristics was performed based on previous information [9, 54–56].

### Phytochemical investigation

#### Extraction procedure

Air-dried powdered aerial parts of each species (40 g) were separately extracted using 70% ethanol (500 mL) via sonication for 2 h at 60 °C, then filtered over charcoal to yield eight aqueous ethanol extracts. Each extract was individually concentrated under reduced pressure at 50 °C to produce a dried extract.

#### LC-ESI-MS/MS analysis and data processing

The phytochemical constituents of the eight aqueous ethanol extracts of *Ipomoea* species were analyzed by LC-ESI-MS/MS, through an Exion LC AC system for separation and a SCIEX Triple Quad 5500 + MS/MS system equipped with electrospray ionization for detection. The investigation was performed using a C18 column (2.1 × 150 mm^2^, 2.7 μm) at 40 °C, with a 5 µL injection volume and a flow rate of 0.3 mL/min. The mobile phases consisted of solvent A: ammonium formate (5 mM, pH 8) and solvent B: acetonitrile (HPLC grade), using a gradient elution from 5% to 100% B over 30 min (Supplementary File). MS/MS analysis was conducted in negative ion mode over a mass range of 100 to 1000 Da, using EMS-IDA-EPI acquisition. Key parameters included an ion spray voltage of -4500 V and source temperature of 500 °C (Supplementary File) [57]. Peak detection and spectrum interpretation were performed with PeakView^®^ 1.2 Software (SCIEX, Framingham, MA, USA) (Supplementary Fig. S1).

#### Molecular network and metabolites annotation

Raw data were converted into open-source files (mzML format) using the MSConvert tool (ProteoWizard Software Foundation, Version 3.0.1933, USA) and subsequently uploaded to the online workflow on the GNPS website (http://gnps.ucsd.edu) via WinSCP [57]. Due to GNPS platform limitations, a single classical molecular networking job can include a maximum of six sets (G1-G6). As our dataset comprised eight samples plus one blank, which exceeded this limit, the analysis was split. The (mzML) datasets of the four wild species (*I. cairica*, *I. carnea*, *I. eriocarpa*, and *I. imperati*) were grouped with the blank file in one network job to create the negative molecular network of wild species (MNW) (Supplementary Fig. S2). In contrast, the datasets of the four cultivated species (*I. batatas*, *I. ochracea*, *I. pes-caprae*, and *I. tricolor*) were analyzed with the blank in a separate network job to construct the negative molecular network of cultivated species (MNC) (Supplementary Fig. S3). This approach allowed accurate network construction, appropriate blank subtraction, and reliable comparison within each ecological group. For each job, the applied parameters were as follows: parent mass tolerance (2.0 Da), a fragment ion tolerance (0.05 Da), a cosine score (0.65), and minimum shared fragments (4) [58]. The two networks were then visualized using Cytoscape version 3.9.1.

In addition to metabolite dereplication through the GNPS platform, the annotation process was further validated through multiple complementary approaches. For putatively identified metabolites, MS data and retention times were cross-referenced with published literature and searched against selected natural product databases (Supplementary File). Where possible, compounds were confirmed by comparison with authentic reference standards (Table 3).

### Data analysis

The recorded macro- and micromorphological characters (Table 2) were coded as multistate characters (exist in two or more distinct states**)**, whereas the phytochemical ones were coded as binary state characters (only two states, present/absent) (Supplementary Table S1). Past 4.03 free software (https://past.en.lo4d.com/windows) was employed to construct a dendrogram using Euclidean coefficient similarity index and UPGMA algorithm clustering [59].

## Results

### Morphological investigation

**Fig. 2:**
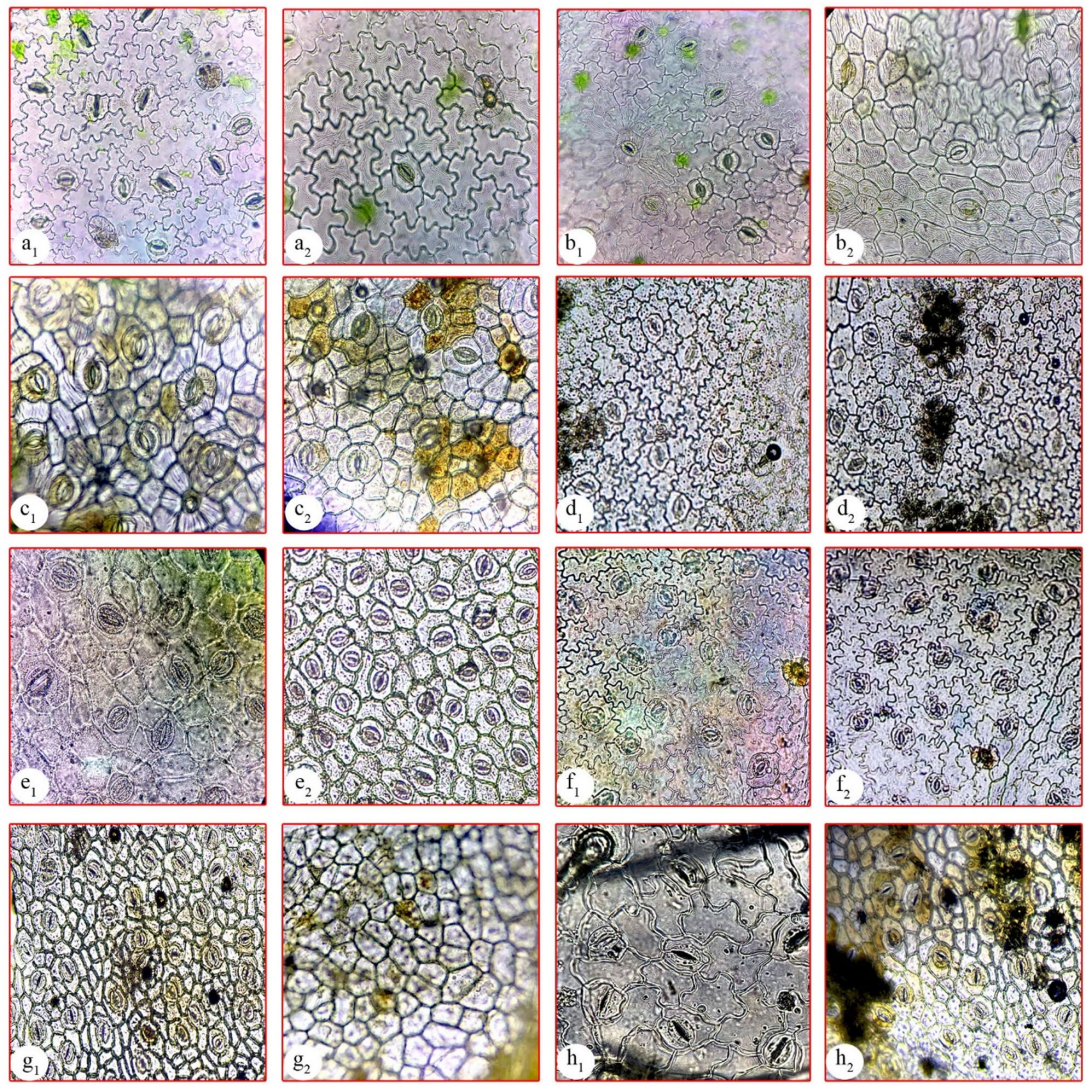
Major aspects of the lamina epidermis using a light microscope (LM; X = 10) showing cell shapes and stomata types of the studied species. **a**: *Ipomoea batatas*, **b**: *I. cairica*, **c**: *I. carnea*, **d**: *I. eriocarpa*, **e**: *I. imperati*, **f**: *I. ochracea*, **g**: *I. pes-caprae*, **h**: *I. tricolor*, 1: abaxial surface, 2: adaxial surface

**Fig. 3:**
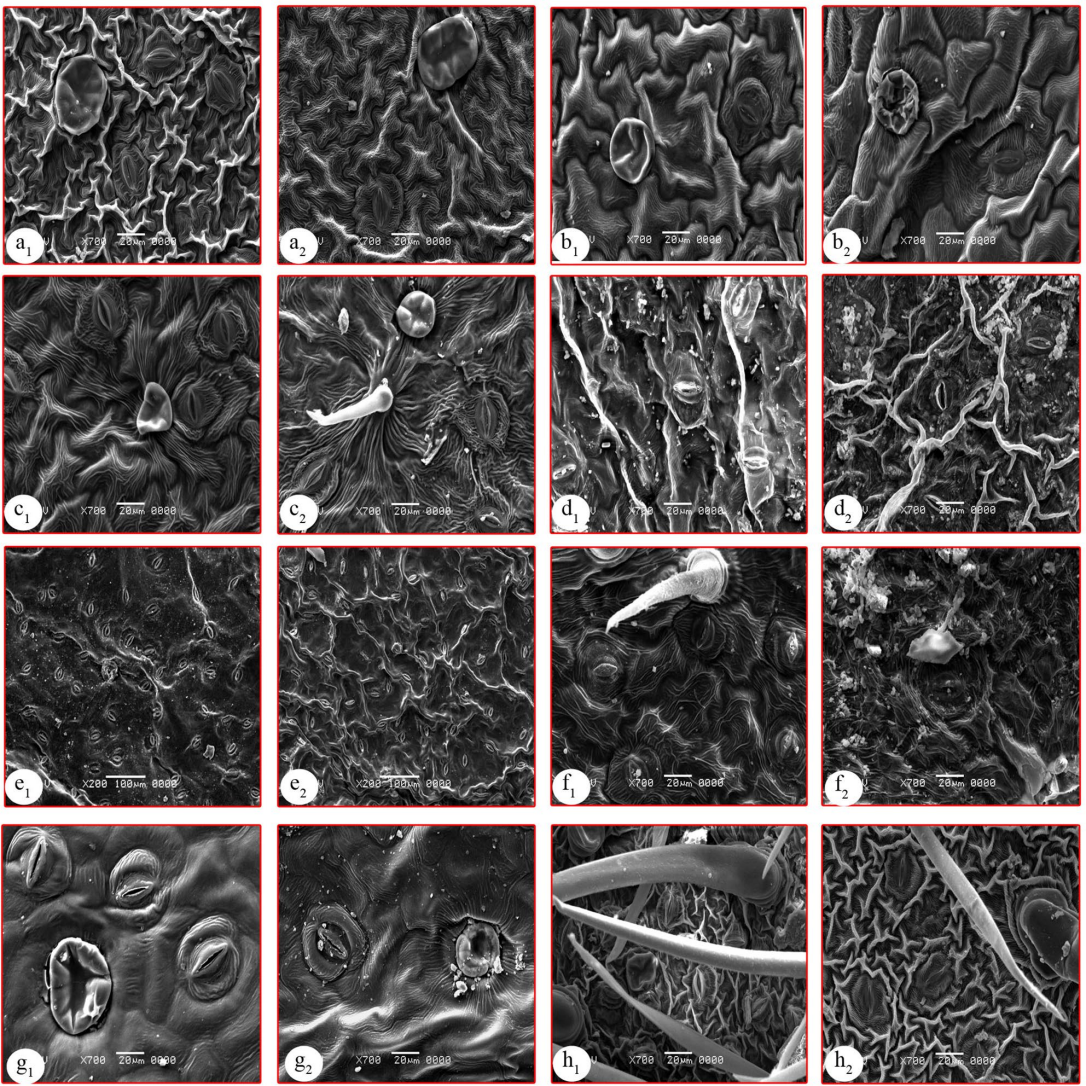
Major aspects of the lamina epidermis using a scanning electron microscope (SEM) showing cell shapes and stomata types of the studied species. **a**: *Ipomoea batatas*, **b**: *I. cairica*, **c**: *I. carnea*, **d**: *I. eriocarpa*, **e**: *I. imperati*, **f**: *I. ochracea*, **g**: *I. pes-caprae*, **h**: *I. tricolor*, 1: abaxial surface, 2: adaxial surface

Many macro- and micromorphological characters were examined, including explicit diagnostic traits at the species levels, such as plant habit, leaf composition, lamina shape, nectary gland, trichome type, vascular system, pith type and secretory cells. The examined characters were recorded in Table 2 and illustrated in Figs. 1, 2 and 3.

**Table 2:** Macro- and micromorphological characters and data matrix of the studied species

Characters, character states, and their codes	Studied species							
	a	b*	c*	d*	e*	f	g	h
Macromorphological characters								
Growth habit: Shrub (1), Herb (2)	2	2	1	2	2	2	2	2
Duration: Biennial (1), Perennial (2)	2	2	2	1	2	2	2	2
Stem strength: Erect (1), Weak (2)	2	2	1	2	2	2	2	2
Leaf insertion: Petiolate (1), Sessile (2)	1	1	1	1	1	1	1	1
Leaf composition: Simple (1), Palmatifid (2), Palmatipartite (3)	2	3	1	1	1	1	1	1
Leaf arrangement: Alternate (1), Opposite (2)	1	1	1	1	1	1	1	1
Lamina shape: Cordate (1), Ovate (2) Orbicular (3), Obong ovate (4), Sub-hastate (5)	1	3	1	5	4	2	3	1
Lamina margin: Entire (1), Serrate (2)	1	1	1	1	1	1	1	1
Lamina apex: Acute (1), Acuminate (2), Notched (3), Emarginate (4)	1	1	1	2	3	2	4	1
Lamina firmness: Thin (1), Fleshy (2)	1	1	1	1	2	1	2	1
Lamina texture: Glabrous (1), Pubescent (2), Hairy (3)	1	1	3	2	1	1	1	2
Lamina veins: Pinnate (1), Palmate (2)	2	2	1	1	1	1	1	2
Nectary glands in lamina base: Present (1), Absent (2)	2	2	2	2	2	2	1	2
Micromorphological characters (Stem anatomy; LM)								
Outline: Terete (1), Triangular (2)	1	1	1	1	1	1	2	1
Epidermis: Tangential (1), Radial (2)	1	2	2	1	1	1	1	2
Cuticle: Thin (1), Thick (2)	1	1	2	1	2	1	2	1
Striated cuticle: Present (1), Absent (2)	1	1	1	1	2	2	2	2
Hypodermis: Present (1), Absent (2)	1	1	2	2	2	2	2	2
Trichomes: Glandular (1), E-glandular with multicellular base (2), Glandular & e-glandular with unicellular base (3), Glandular & e-glandular with multicellular base (4), Absent (5)	4	5	3	2	5	3	1	2
Trichome density: Abundant (1), Scarce (2), Absent (3)	2	3	1	2	3	1	2	2
Periderm: Subepidermal (1), Absent (2)	2	2	1	2	1	1	2	2
Cortex: Two types of cells (1), Three types of cells (2)	2	2	2	2	2	1	2	2
Pith cells: Solid (1), Hollow (2)	1	1	2	1	1	1	1	1
Pitted parenchyma in pith: Present (1), Absent (2)	2	2	1	2	2	2	2	2
Xylem vessels distribution: Ring porous (1), Diffuse porous (2)	1	1	2	1	1	1	1	1
Fascicular xylem contents: Vessels, fibers, tracheids& parenchyma (1), Vessels, fibers& parenchyma (2)	1	1	2	2	2	1	1	2
Interfascicular xylem contents: Vessels, fibers& tracheids (1), Fibers (2)	1	1	2	2	1	1	1	1
Horizontal system: Uniseriate fascicular & interfascicular (1), Uniseriate fascicular only (2), Absent (3)	3	3	1	3	2	1	1	1
Secretory system (Schizogenous duct): Abundant (1), Scarce (2)	1	2	1	2	1	1	1	1
Location of schizogenous duct: Cortex & pith (1), Cortex (2)	1	1	1	1	2	1	1	2
Tannineferous cells: Present (1), Absent (2)	2	2	2	2	1	2	1	2
Druses: Abundant (1), Scarce (2)	1	1	1	1	2	1	1	1
Druses location: Cortex, pith& phloem (1), Cortex& pith (2)	1	2	2	1	2	1	1	1
Micromorphological characters (Lamina anatomy; LM)								
Trichomes: Glandular (1), E-glandular with multicellular base (2), Glandular & e-glandular with unicellular base (3), Glandular & e-glandular with multicellular base (4), Absent (5)	3	1	3	2	5	2	1	4
Epidermis in midrib: Radial (1), Tangential (2)	1	1	1	1	1	2	1	1
Mesophyll type: Isolateral (1), Dorsiventral (2)	2	2	2	2	1	2	1	2
Extended palisade in midrib continuity: Extended not connected (1), Extended & connected adaxial (2), Extended & connected abaxial (3)	1	2	1	1	3	1	1	1
Outline adaxial: Concave (1), Ridged (2)	2	2	2	2	1	2	1	2
Secretory system (Schizogenous duct): Present (1), Absent (2)	2	2	1	2	1	1	1	1
Mid vein vascular bundle: Crescent shape (1), Horseshoe shape (2)	1	1	2	1	1	1	1	2
Druses: Abundant (1), Scarce (2)	2	2	1	1	1	1	1	1
Micromorphological characters (Lamina epidermis; LM &SEM)								
Cells shape (Ab-/Adaxial): Irregular/same (1), Polygonal/same (2), Irregular/polygonal (3)	1	3	2	1	2	1	2	1
Anticlinal wall shape (Ab-/Adaxial): Sinuous/ same (1), Straight/same (2), Wavy/straight (3), Curved/ same (4)	1	3	2	1	2	1	2	4
Stomata type: Paracytic (1), Paracytic & anisocytic (2)	1	1	2	1	1	2	1	2
Sculpture (Ab-/Adaxial): Ruminate/same (1), Alveolate/ favulariate (2), Rugose/ same (3), Weak pusticulate/ pusticulate-weak reticulate (4), Ruminate-rugose/ ruminate (5), Weak reticulate/ reticulate -areolate (6), Ruminate/ rugose (7)	1	1	2	3	4	5	6	7
Anticlinal wall thickness; SEM (Ab-/Adaxial): Thin/ same (1), Thick/ same (2)	1	1	1	2	2	2	1	1
Anticlinal wall elevation (Ab-/Adaxial): Depressed/ same (1), Raised/ same (2)	1	1	1	1	2	1	1	1
Periclinal wall elevation (Ab-/Adaxial): Depressed/ same (1), Raised/ same (2)	2	2	2	2	1	2	2	2
Periclinal wall surface (Ab-/Adaxial): Striate/ same (1), Granulate/ same (2)	1	1	1	1	2	1	1	1
Periclinal wall granulation (Ab-/Adaxial): Present (1), Absent (2)	2	2	2	1	1	1	2	1
Periclinal wall striation (Ab-/Adaxial): Present (1), Absent (2)	2	1	1	2	2	2	1	2

a: *Ipomoea batatas*, b: *I. cairica*, c: *I. carnea*, d: *I. eriocarpa*, e: *I. imperati*, f: *I. ochracea*, g: *I. pes-caprae*, h: *I. tricolor*. Stem cortex: Two types of cells (collenchyma and parenchyma), Three types of cells (collenchyma, parenchyma, and sclerenchyma), (*): wild taxa, (LM): light microscope, (SEM): scanning electron microscope

### Phytochemical investigation

#### LC-ESI-MS/MS metabolites profiling and molecular networking

**Fig. 4:**
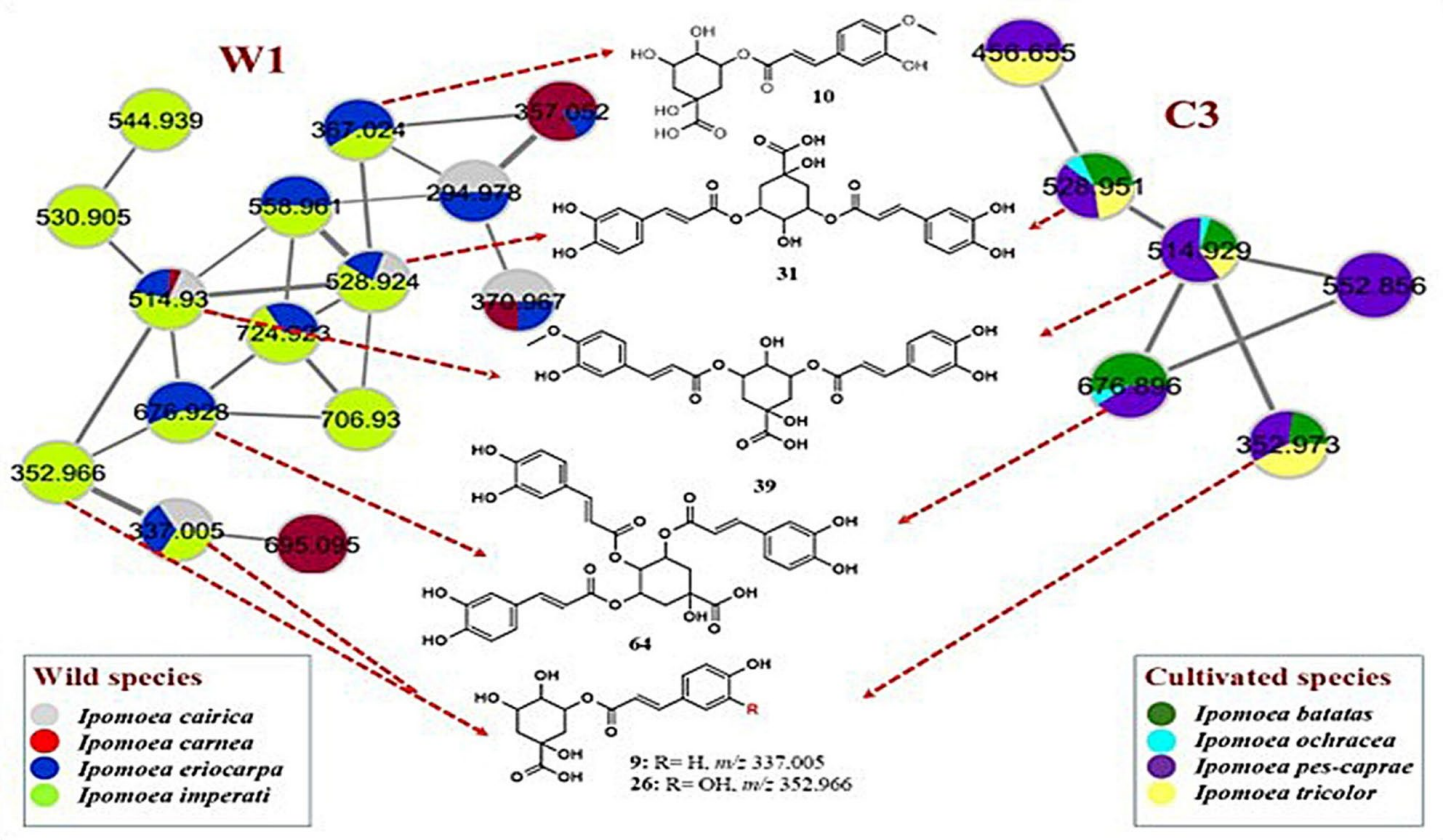
The significant clusters (W1 and C3), representing the distribution of hydroxycinnamic acid esters within the studied *Ipomoea* species (wild and cultivated species, respectively)

**Fig. 5:**
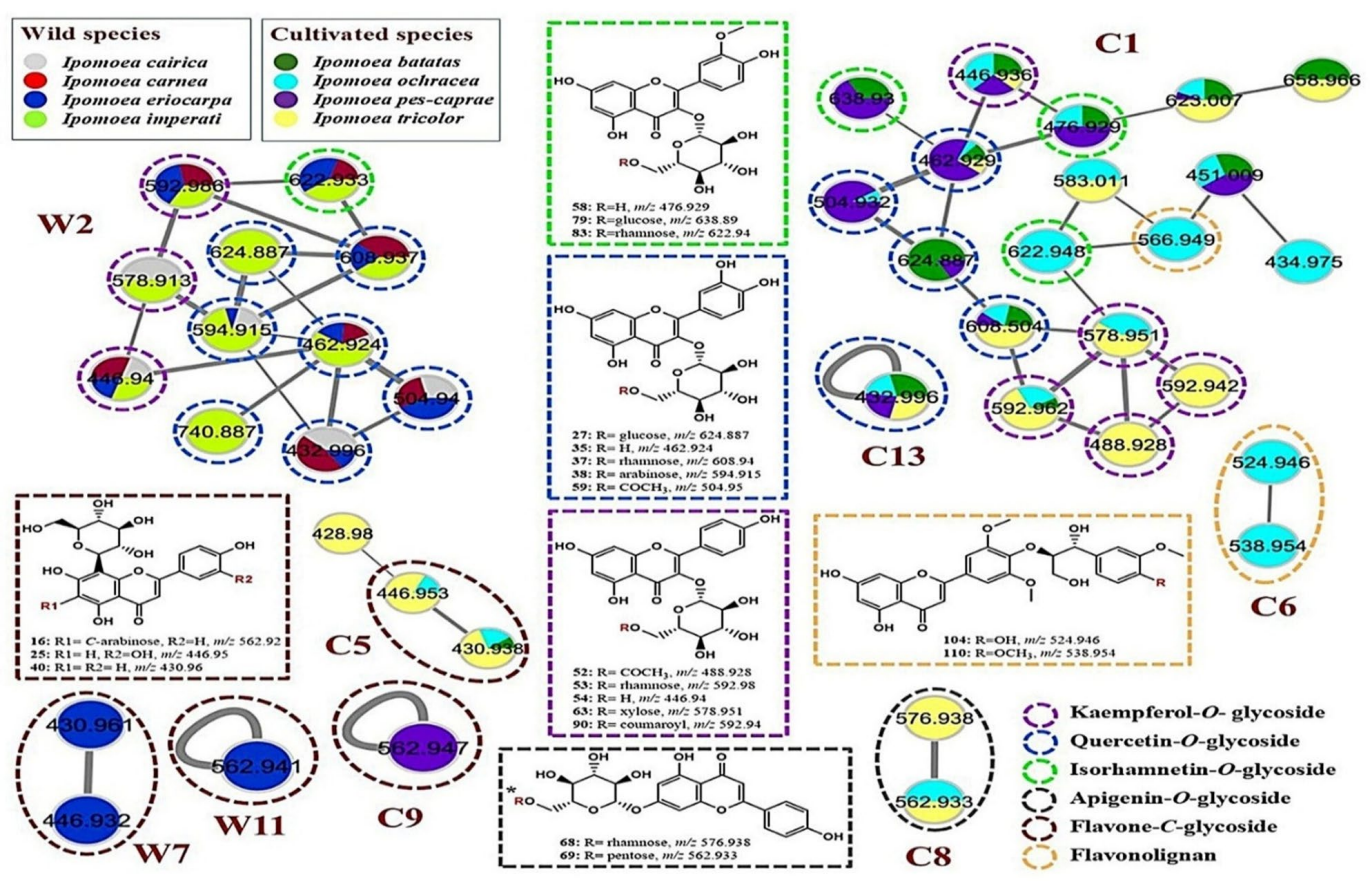
The significant clusters (W2) and (C1, C3, & C8), represent the flavonoids-*O*-glycosides, and (W7 & W11) and (C5 & C9), represent the distribution of flavonoids-*C*-glycosides within the studied *Ipomoea* species (wild and cultivated species, respectively), with the exclusive existence of flavolignans (C6) in *I. ochracea*

**Fig. 6:**
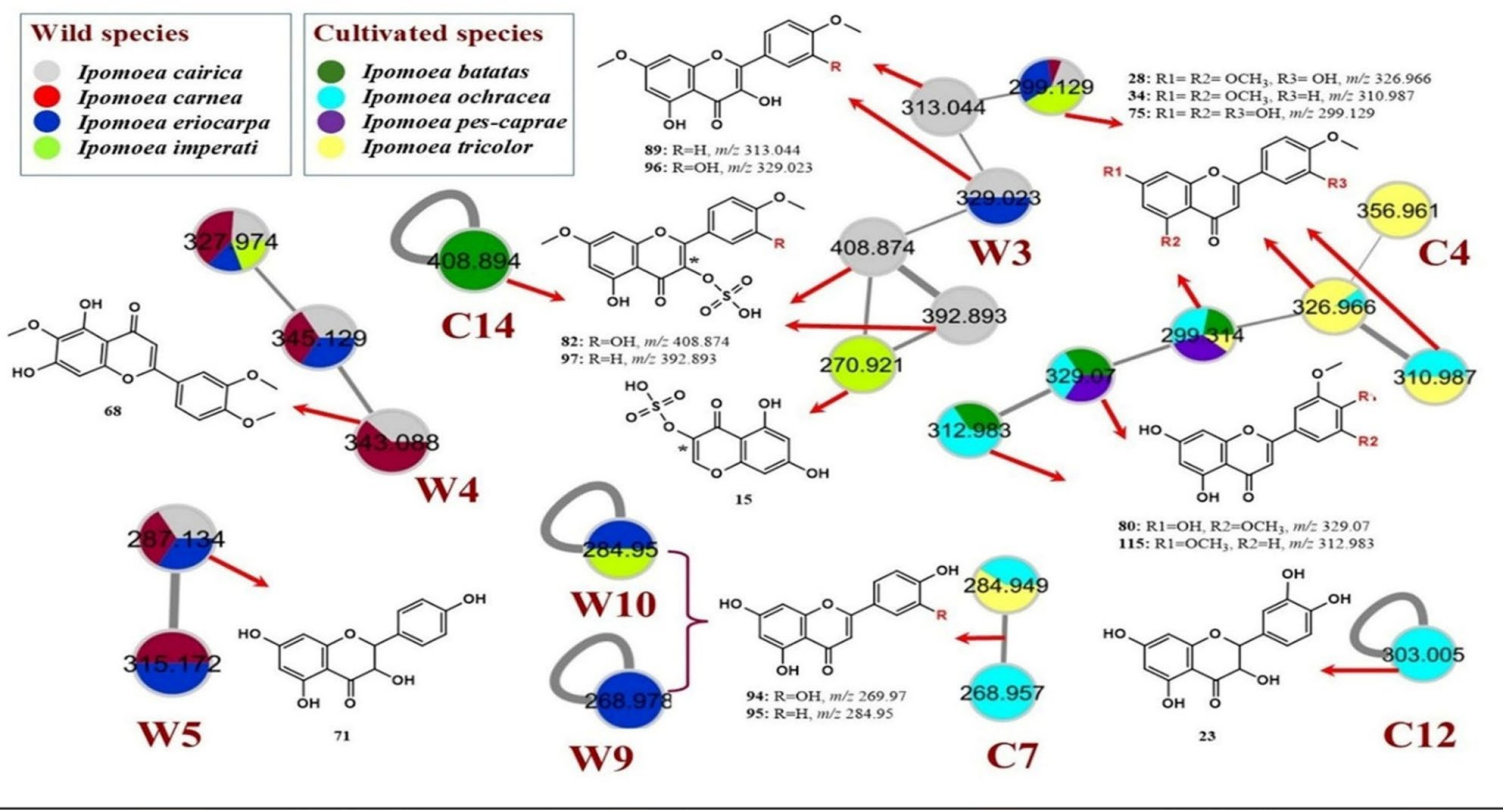
The significant clusters (W3, W4, W5, W9 & W10) and (C4, C7, & C12), represent the flavonoid aglycones within the studied *Ipomoea* species (wild and cultivated species, respectively), with the exclusive existence of sulphated flavonoid aglycones (a part of W3) and (C14) in *I. cairica* and *I. batatas*, respectively

LC-ESI-MS/MS analysis, in the negative ionization mode, was imperative to comprehensively characterize and compare the metabolite compositions of the eight selected *Ipomoea* species. The overlaid base peak chromatograms of the eight extracts exhibited some differences, especially at the *Rt* range of (2–15 min) (Supplementary Fig. S1). In performance LC-MS processing, metabolites annotation was administered, guiding *Rt*, precursor ions, and MS fragmentation pattern. For a more accurate interpretation, two molecular networks were created: MNW (Supplementary Fig. S2) and MNC (Supplementary Fig. S3) to visualize the chemical diversity of the eight *Ipomoea* species. With the aid of GNPS libraries, possible connections between each MS^2^ spectrum were established, in this manner, enabling additional annotation of related structures. In the constructed MNW, a total 417 nodes including 213 were grouped in 44 clusters (at least clusters with two nodes), and 204 self-looped nodes (Supplementary Fig. S2), comprising interesting phenolic structures such as hydroxycinnamic acid esters (W1) (Fig. 4), and flavonoid-*O*-glycosides (W2), flavonoid-*C*-glycosides (W7) (Fig. 5), flavonoid aglycones (W3, W4, W5 and W9, and W10) (Fig. 6). On the other hand, the MNC includes 414 nodes (218 connected nodes and 196 self-looped nodes). The connected nodes are grouped in 55 clusters (Supplementary Fig. S3), involving remarkable clusters of various chemical groups: hydroxycinnamic acid esters (C3) (Fig. 4), flavonoid-*O*-glycosides (C1 and C13), flavonoid-*C*-glycosides (C5 and C9), flavonolignans (C8) (Fig. 5), flavonoid aglycones (C4, C7, C12, and C14) (Fig. 6), and fatty acid derivatives (C2). Both molecular networks are displayed as a pie chart to replicate the relative abundance of each ion in the studied *Ipomoea* extracts. A total of 132 compounds were characterized, including 107 secondary metabolites (68 flavonoids, 34 phenolic acids, 4 coumarins, and one anthocyanin) and 25 primary metabolites (16 fatty acid derivatives, five amino acids, two saccharides, two phospholipids, and one organic acid) (Table 3). Among them, extensive chemotypes such as phenolic acids and flavonoids highlight the chemotaxonomic differentiation of the investigated *Ipomoea* species.

**Table 3:** Tentative identification of phytochemical constituents in *Ipomoea* species extract

No.	Tentative identification	RT (min)	[M-H]^−^	MS/MS	a	b*	c*	d*	e*	f	g	h	Ref
Secondary metabolites													
	Phenolic acids and derivatives												
	**Hydroxycinnamic acids and derivatives**												
5	*p*-Coumaric acid ^€, $, #, a^	1.68	163.01	135, **119**	+	+	+	+	+	+	+	+	[60]
6	Caffeic acid ^$, #, a, c, g^	1.76	179.01	163, **135**, 119, 107	+	+	+	+	+	+	+	+	[61]
9	Coumaroyl quinic acid	2.09	337.002	**191**, 173, 163, 145, 127, 119, 111	-	+	-	+	+	+	+	-	[60]
10	Feruloyl quinic acid ^#, a^	2.11	367.01	193, 191, **173**, 134	-	-	-	+	+	-	-	-	[62]
14	Caffeic acid-*O*-glucoside	2.69	340.98	179, 161, 149, 135, 109	+	-	-	-	-	-	+	+	[63]
19	Coumaric acid isomer ^€, $, #, a^	4.69	163.01	135, **119**, 100	+	+	+	+	+	+	+	+	[61]
21	Dimethoxy cinnamic acid di-*O*-hexoside	2.59	530.91	369, 339, **207**, 191, 179, 135	-	-	-	-	+	-	-	-	
26	Caffeoyl quinic acid ^€, #, a^	3.92	352.969	**191**, 177, 163, 111	+	-	-	-	+	-	+	+	[63]
30	Dimethoxy cinnamic acid di-*O*-hexoside isomer	5.18	530.91	369, 339, **207**, 191, 179, 135	-	-	-	-	+	-	-	-	
31	Feruloyl caffeoyl quinic acid ^€, #, a^	5.21	528.927	353, 191, 179, 173, 135	+	+	-	+	+	+	+	+	[64]
33	Caffeic acid isomer ^$, #, a, c, g^	5.77	179.01	151, 147, **135**, 119, 117, 107	+	+	+	+	+	-	+	+	[61]
39	Dicaffeoyl quinic acid ^#, a, b, g^	5.89	514.93	515, 353, 191, 179, **173**, 135	+	+	+	+	+	+	+	+	[60]
44	N-Feruloyltyramine ^#, a^	6.17	312.25	297, 275, 253, 193, 190, 179, 161, **148**, 135, 109	+	+	+	+	+	+	+	+	[65]
50	Caffeoyl coumaroyl quinic acid	6.44	498.93	337, 191, 179, **173**, 163, 135	+	-	-	-	+	+	+	-	
51	Caffeoyl sinapoyl quinic acid	6.65	**558.95**	397, 223, 191, 173, 161, 149	+	-	-	+	+	-	+	+	[61]
60	Dimethoxy cinnamic acid tri-*O*-hexose	7.17	692.89	531, 369, 339, **207**, 191, 179, 135	+	-	-	-	+	-	-	-	
64	Tricaffeoyl quinic acid ^#, a^	7.41	676.89	**515**, 353, 191, 179, 173, 135	+	-	-	+	+	+	+	-	[61]
66	Dicaffeoyl quinic acid isomer ^#, a, b, g^	7.65	514.93	515, 353, 191, 179, **173**, 135	+	+	+	+	+	+	-	+	[60]
73	N-Caffeoyltyramine	8.40	297.95	179, 178, 161, **135**	+	+	-	+	-	-	+	-	
81	N-Coumaroyltyramine ^#, a^	9.17	282.03	163, 145, **119**, 107	+	+	-	+	+	+	+	+	[31]
84	N-Feruloyltyramine isomer ^#, a^	9.73	312.268	297, 253, 193, 190, 179, 161, **148**, 135, 109	+	+	+	+	+	+	+	+	[65]
85	N-Sinapoyltyramine	9.77	342.326	327, 312, 203, 190, 178, 161, **148**, 135, 109	-	-	-	+	+	-	-	-	[66]
99	Coumaraldehyde ^€^	10.29	145.08	**117**, 101	+	-	-	-	-	+	+	+	[67]
101	Sinapaldehyde	10.49	207.12	179, 161, **135**	-	-	-	-	-	-	-	+	[67]
103	Ferulic acid-*O*-trihexoside	10.77	679.02	185, **193**, 178, 175, 149, 135, 177	-	-	-	-	-	-	-	+	
105	Dimethoxy cinnamic acid	10.98	207.02	177, 163, 147, **133**, 105	-	-	-	-	-	-	-	+	
108	Ferulic acid-*O*-sulphate	11.77	272.92	**193**, 179, 161, 149, 133, 117	+	+	+	+	+	+	+	+	
109	N-Sinapoyl putrescine	11.83	293.11	223, 205, 195, 179, 177, **171**	-	-	-	+	-	-	-	+	[68]
116	Ferulic acid ^$, #, a^	13.45	193.01	179, 161, 149, **133**	+	-	-	-	-	-	+	-	[61]
	**Hydroxybenzoic acids and derivatives**												
8	Dihydroxybenzaldehyde ^€, #, g^	1.99	136.952	**121**, 109	+	+	+	+	+	+	+	+	[69]
17	p-Hydroxybenzaldehyde ^€, $^	3.52	**120.989**	108	+	+	+	+	+	+	+	+	[61]
22	Dihydroxybenzoic acid ^€^	2.92	152.98	135, 117, **109**	+	+	-	+	+	+	-	+	[61]
43	Vanillic acid ^€, $, #, a, b^	6.15	167.01	153, 123, **109**	+	+	+	+	+	-	+	+	[61]
62	Dihydroxybenzoic acid-*O*-hydroxybenzoyl glucoside/ Parmentin A ^€^	7.33	434.975	315, 297, 153, **137**, 109	-	-	-	-	-	+	-	-	[70]
	**Flavonoids**												
	**C-glycosyl flavonoids**												
12	Luteolin methyl ether 8-*C-*glucoside	2.29	460.91	**371**, 341	+	-	-	-	+	+	+	+	[71]
16	Apigenin 6-*C-*arabinose-8-*C*-glucose/ Corymboside ^€, $^	3.46	562.92	503, 473, 443, 425, 383	-	-	-	+	-	-	+	-	[60]
18	Eriodictyol 6-*C-*glucoside	3.77	**448.92**	359, 329	-	-	-	-	-	-	-	+	[72]
20	Apigenin 6-*C-*glucose *−* 8-*C*-arabinose/ Schaftoside ^€, $^	4.75	562.94	503, 473, 443, 425, 383	-	-	-	+	-	-	+	-	[60]
24	Naringenin 6-*C*-glucoside	3.55	432.96	343, 313, 271, 193, 119	-	-	-	-	-	-	-	+	[72]
25	Orientin	3.91	446.95	429, 357, 327, 297	-	-	-	+	-	+	-	+	[71]
29	Isoorientin ^#, e^	5.02	446.95	429, 357, 327, 297	-	-	-	+	+	+	-	+	[71]
40	Vitexin ^€, $, #, e^	6.07	430.96	341, **311**	+	-	-	+	-	+	-	+	[72]
45	Vitexin 7-*O*-glucoside/ Saponarin ^€, $^	6.19	**592.98**	431, 341, 311	+	-	-	+	-	+	-	+	[72]
	**Flavonoid-** ***O*** **-glycosides**												
27	Quercetin 3-*O*-sophroside ^€, #^	4.58	**624.88**	463, 301, 300, 271, 179, 173 161, 151, 135, 109	+	-	-	-	+	-	+	-	[73]
32	Quercetin 3-*O*-arabinosyl rutinoside	5.28	**740.887**	609, 301, 300, 179, 151	-	-	-	-	+	-	-	-	
35	Quercetin 3-*O*-glucoside ^€, $, #, a^	5.91	462.96	301, **300**, 271, 255, 179, 151, 107	+	+	+	+	+	+	+	+	[61, 35]
37	Quercetin 3-*O*-rutinoside/ rutin ^$, #, a, c, e^	5.57	**608.94**	301, 300, 283	+	+	+	+	+	+	+	+	[60]
38	Quercetin 3-*O*-arabinosyl glucoside/ Quercetin-3-*O*-vicianoside ^€, $^	5.49	**594.91**	433, 301, 300, 271	+	+	+	-	+	+	-	+	[62]
41	Kaempferol 3-*O*-glucoside 7-*O*-rhamnoside ^$, #, a^	6.08	592.98	**447**, 431, 285, 255	-	-	-	-	-	-	-	+	[61]
42	Quercetin-*O*-glucosyl rhamnoside	6.09	**608.96**	447, 301, 300, 229	+		-	-	-	+	+	-	
46	Kaempferol 3-*O*-glucoside-xyloside ^€^	6.26	578.95	**447**, 417, 285, 284	-	+	-	-	+	+	-	+	[62]
47	Luteolin methyl ether 6-*C-*glucoside	6.28	460.92	361, 353, **341**, 299, 241	-	-	-	-	-	-	-	+	[71]
48	Quercetin 3-*O*-arabinoside ^€, b^	6.35	432.93	301, **300**, 271, 255	+	+	+	+	-	+	+	+	[74]
53	Kaempferol 3-*O*-rutinoside ^€, $^	6.68	**592.98**	**285**, 284, 257, 229	-	+	+	+	+	+	-	+	[61]
54	Kaempferol 3-*O*-glucoside ^€, $^	6.73	446.94	285, **284**, 255, 227, 163, 151, 135	+	+	+	+	+	+	+	+	[61]
58	Isorhamnetin 3-*O*-glucoside ^€, $^	6.98	476.929	315, **314**, 301, 271, 243, 151	+	-	+	+	+	+	+	-	[61]
63	Kaempferol 3-*O*-arabinosyl -glucoside/ Kaempferol 3-*O-*vicianoside ^€, $^	7.35	**578.97**	447, 285, 284, 153	-	-	-	-	-	-	-	+	[63]
65	Trihydroxy-trimethoxy flavone-*O*-glucoside	7.44	520.951	359, **329**, 313, 298, 193, 178, 161, 135	+	-	-	-	-	+	-	+	
68	Apigenin 7-*O*-rutinoside ^€, $^	7.83	576.94	**269**, 227, 225, 159, 135	-	-	-	-	-	-	-	+	[75]
69	Apigenin 7-*O*-pentosyl glucoside ^€^	7.99	562.93	431, **269**, 227, 179, 161, 133	-	-	-	-	-	+	-	+	
70	Apigenin 7-*O*-glucoside ^€, $, #, c^	8.12	**430.98**	269, 241, 227, 167, 151, 117	+	+	-	+	+	+	+	+	[60]
72	Monohydroxy-hexamethoxy flavone-*O*-glucoside	8.36	578.99	**417**, 401, 387, 373, 359, 327	+	+	-	-	-	-	-	-	
76	Chrysoeriol-*O-*glucoside ^#, a^	8.52	**460.91**	445, 299, 283, 255, 161, 133	+	-	-	-	-	+	+	+	
77	Quercetin 3-*O*-glucoside-7-*O*-rhamnoside ^$^	8.61	608.94	**463**, 447, 301, 300, 285, 151	-	-	-	-	-	-	-	+	[62]
79	Isorhamnetin 3-*O*-sophroside ^$^	8.79	**638.89**	477, 315, 299, 285, 271, 259, 227, 151	-	-	-	-	+	-	+	-	[74]
83	Isorhamnetin 3-*O*-rutinoside ^$^	9.45	622.95	477, 461, 315, 299, 285, 161, 137	-	-	+	+	+	+	-	-	[63]
91	Eriodictyol 7-*O-*rutinoside ^a^	9.35	**596.98**	287	-	-	-	+	+	-	-	-	
111	Chrysoeriol-di-*O-*glucoside ^€, #, a^	12.05	623.001	461, 299, 297, 283	+	+	+	+	+	+	+	+	
	**Flavonoid** ***O*-acyl glycosides**												
49	Kaempferol 3-*O*-feruloyl pentoside ^€^	6.41	**592.97**	417, 399, 285, 284, 193, 179, 161, 153, 135, 108	-	-	-	-	-	-	-	+	
52	Kaempferol 7-*O*-acetyl glucoside	6.66	488.928	447, **285**, 284, 179, 165, 161, 135	-	-	-	-	-	-	-	+	
55	Kaempferol 3-*O*-malonyl glucoside ^€^	6.75	532.88	**489**, 447, 285, 284	-	-	-	-	-	-	-	+	[62]
56	Quercetin 3-*O*-feruloyl pentoside	6.76	**608.94**	433, 415, 301, 300, 193, 179, 161, 152, 135, 108	-	-	-	-	-	-	-	+	
59	Quercetin 3-*O*-acetyl glucoside	7.04	504.95	463, 301, **300**, 271, 255, 151, 137	-	+	+	+	+	+	+	-	[62]
90	Kaempferol 3-*O*-coumaroyl glucoside ^€^	9.22	**592.94**	285, 284, 447, 255, 163, 145	-	-	-	-	-	-	-	+	
106	Kaempferol 3-*O*-caffeoyl glucoside ^€, $, #^	11.26	**608.94**	447, 285, 284, 179, 161, 135	-	-	-	-	-	-	-	+	
	**Flavonoid aglycones**												
23	Taxifolin ^€, $^	3.36	**303.004**	285, 223, 163, 137, 125, 113	-	+	+	+	-	+	-	-	[61]
28	Monohydroxy trimethoxy flavone ^€^	4.98	**326.966**	299, 284, 175, 163, 149, 133, 109	-	-	-	-	-	+	-	+	
34	Trimethoxy flavone ^€^	4.98	310.987	**283**, 269, 224, 163, 149, 135, 117	-	-	-	-	-	+	-	+	
57	Dihydromyricetin/ Ampeloptin ^€, $^	6.84	**319.03**	287, 255, 165, 137, 123, 108	-	+	-	-	-	-	-	-	
71	Dihydrokaempferol/ Aromadendrin ^€^	8.28	287.13	269, 241, 225, 163, 155, 137, 108	-	+	+	+	-	+	-	-	
74	Isorhamnetin ^€, $, #, a^	8.41	315.01	299, 285, 271, 259, 229, **163**, 151, 121	+	-	+	+	-	-	-	-	[74]
75	Chrysoeriol ^€, $, #^	8.48	**299.02**	283, 271, 255, 227, 161, 133	+	+	+	+	+	+	+	+	[72]
78	Quercetin ^€, $, #, a, g, h^	8.88	300.95	283, 269, 255, 229, 179, **151**, 121, 108	-	-	-	+	+	-	-	-	[61]
80	Tricin ^€, $^	9.15	329.07	314, **299**, 285, 271, 255, 227	+	-	-	-	-	+	+	-	[61]
82	Ombuin-3-sulphate ^#, b^	9.31	408.874	329, **314**, 313, 299, 271, 243, 225	+	+	-	-	-	-	-	-	[66]
86	Dihydroxy trimethoxy flavone/Eupatilin ^€, $^	9.87	343.088	328, **313**, 298, 285, 269, 257, 254, 241	+	+	-	-	-	-	-	-	
88	Naringenin ^€, $, #, a^	9.02	270.982	253, 227, 187, 177, 151, **119**, 107	+	+	-	-	-	-	-	-	[61]
89	Kaempferol dimethyl ether	9.14	313.044	**298**, 283, 271, 255, 227	-	+	-	-	-	-	-	-	
92	Myricetin ^€, $, #, a^	9.51	317.11	302, 301, 210, 167	+	+	-	+	-	+	-	-	[61]
94	Apigenin ^€, $, #, a^	9.79	**268.97**	227, 225, 151, 149, **117**, 107	-	-	-	+	-	+	-	-	[74]
95	Luteolin ^€, $, #^	9.97	284.95	267, 241, 175, 151, **133**, 107	-	-	-	+	+	+	-	+	[75]
96	Quercetin dimethyl ether/ ombuin ^€^	10.10	329.023	314, **299**, 271, 243, 227, 161, 135	-	+	-	+	-	-	-	-	
97	Kaempferol dimethyl ether-3-sulphate	10.17	392.893	313, **298**, 283, 271, 255, 227	-	+	-	-	-	-	-	-	
102	Kaempferol ^€, $, #, a, e^	10.57	**284.97**	257, 229, 151, 133	+	-	+	-	-	-	-	+	[62]
107	Rhamnetin-3-*O*- sulphate ^#, b^	10.17	**395.05**	315, 300, 269, 255, 227, 165	+	+	-	-	-	-	-	-	
115	Luteolin dimethyl ether / Velutin ^€, $, #^	13.41	**312.983**	298, 283, 255, 241, 227	+	+	-	-	-	+	-	-	[76]
120	Tetrahydroxy dimethoxy flavone	14.45	345.01	331, 315, **301**, 286, 271, 225, 161, 137	+	+	+	+	-	+	-	-	
	**Flavonolignans**												
87	Tricin -*O*-acetyl guaiacyl glycerol	10.83	566.97	**329**, 299, 193, 178, 137	-	-	-	-	-	+	-	-	[77]
104	Tricin -*O*-guaiacyl glycerol	10.86	524.946	**329**, 314, 299, 195, 165, 150	-	-	-	-	-	+	-	-	[78]
110	Tricin -*O*-dimethoxy phenyl glycerol	11.88	538.954	**329**, 314, 299, 275	-	-	-	-	-	+	-	-	[77]
	**Coumarins**												
15	Scopoletin-*O*-sulphate	2.99	270.921	191, **176**, 148	-	-	-	-	+	-	-	-	
36	Scopoletin ^€, $, #, a, b, c, h^	5.48	190.996	**177**, 148, 120, 104	+	+	+	+	+	+	-	-	[62]
93	Trihydroxy coumarin	9.59	193.08	161, 149, **133**	-	+	+	+	+	-	-	-	[61]
98	Dihydroxycoumarin/ Esculetin ^€, $, #, a, g^	10.22	176.956	145, **117**	+	-	-	-	-	-	+	+	[61]
	**Anthocyanins**												
61	Cyanidin 3-*O*-hexose	7.23	449.98 ^@^	288, 287, 286, 151	-	-	-	-	-	-	-	+	[72]
Primary metabolites													
	**Amino and organic acids**												
1	Arginine ^#, a^	0.73	173.06	155, 137, **131**, 113	+	+	+	+	+	+	+	+	[61]
2	Citric acid ^€, #, a^	0.91	191.01	**111**	+	+	+	+	+	+	+	+	[61]
3	Valine ^#, a, b^	1.4	**116.08**	99	+	+	+	+	+	+	+	+	[61]
4	Asparagine ^#, b^	1.65	131.01	115, **113**, 100	-	+	-	+	-	-	-	-	[62]
7	Leucine/Isoleucine ^#, a, c^	1.81	**130.03**	113	+	+	+	+	+	+	+	+	[61]
11	Tryptophan ^#, a, c, h^	2.21	203.03	157, 142, **116**	+	+	+	+	+	+	+	+	[61]
	**Saccharides**												
13	Trihexose/ Raffinose ^#, a^	2.36	502.96	341, 323, 179, **161**, 135	+	+	+	+	+	+	+	+	[79]
100	Tetrahexose/ Stachyose ^€^	10.41	**665.01**	503, 341, 179, 161, 133	+	+	+	+	+	+	+	+	
	**Fatty acids and derivatives**												
67	Trihydroxy-octadecadienoic acid	7.66	327.13	239, 229, **211**, 171, 137	+	+	+	+	+	+	+	+	[61]
112	Oxo octadecatrienoic acid ^€^	12.38	**291.097**	273, 235, 183	+	+	+	+	+	+	+	+	
113	Hydroxy octadecatrienoyl-dihexosyl glycerol	12.48	691.066	415, 397, 293, 275, 235, 195, 125, 113, 101	-	-	-	-	-	-	-	+	
114	Hydroxy octadecatrienoic acid-*O*-dihexoside	12.97	617.088	293, 275, 235, 483, 113, 101	-	-	-	-	-	-	-	+	
117	Hydroxyoctadecatrienoic acid/ Hydroxylinolenic acid ^€^	13.61	**293.12**	275, 223, 185, 125	+	+	+	+	+	+	+	+	[61]
118	Hydroxyoctadecadienoic acid/ Hydroxylinoleic acid ^€^	13.71	**295.18**	277, 259, **171**, 155	+	+	+	+	+	+	+	+	[61]
119	Hydroxyoctadecenoic acid/ Hydroxyoleic acid ^€^	14.42	**297.13**	281, 239, 183	+	+	+	+	+	+	+	+	[61]
122	Hydroxyhexadecanoic acid/ Hydroxypalmitic acid ^€^	14.97	271.11	**225**	+	+	+	+	+	+	+	+	
123	Octadecatrienoyl-dihexosyl glycerol ^€^	15.23	675.092	415, **397**, 277, 253, 235, 161, 125, 101	-	-	-	-	-	-	-	+	
124	Octadecatrienoyl-dihexosyl glycerol isomer ^€^	15.55	721.048 ^@^	415, **397**, 277, 253, 235, 161, 125, 101	+	-	-	-	-	-	-	+	[57]
125	Dihydroxyoctadecenoic acid ^€^	15.79	313.13	297, 281, **267**, 257, 177, 163	-	-	-	+	-	-	+	+	[61]
126	Octadecatrienoic acid-*O*-dihexoside	16.42	647.049 ^@^	277, 233, 179, 113, 101	-	-	-	-	-	-	-	+	
127	Octadecatrienoic acid-*O*-dihexoside isomer	16.71	601.084	277, 233, 179, 113, 101	-	-	-	-	-	-	-	+	
128	Linolenic acid ^#, c, d^	16.83	**277.11**	259, 233, 141	+	+	+	+	+	+	+	+	[72]
129	Apigenin-*O*-pentoside	17.39	401.01	269, 251	+	-	-	-	-	+	-	+	[61]
130	Linoleic acid ^#, c^	20.87	**279.17**	261, 235, 215, 166	+	+	+	+	+	+	+	+	[72]
131	Oleic acid ^#^	23.09	**281.16**	263, 238, 166, 152	+	+	+	+	+	+	+	+	[72]
	**Phospholipids**												
121	Hexadecanoyl-glycero-phospho-inositol	14.58	**571.08**	409, 255, 241, 153	-	-	-	-	+	-	+	-	[67]
132	Linolenoyl-palmitoyl-phosphatidylglycerol ^€^	25.93	**743.19**	277, 255, 153	-	-	-	-	-	-	-	+	

a: *Ipomoea batatas*, b: *I. cairica*, c: *I. carnea*, d: *I. eriocarpa*, e: *I. imperati*, f: *I. ochracea*, g: *I. pes-caprae*, h: *I. tricolor.* The subscript symbols: (*) the wild taxa, (**) [M-H + FA]^−^, (€) compounds identified through GNPS libraries, ($) compounds confirmed by authentic standards, (#) compounds reported before in *Ipomoea* species, (a) compounds reported before in *I. batatas*, (b) compounds reported before in *I. cairica*, (c) compounds reported before in *I. carnea*, (d) compounds reported before in *I. eriocarpa*, (e) compounds isolated before in *I. imperati*, (g) compounds reported before in *I. pes-caprae*, (h) compounds reported before in *I. tricolor.* Bold number labelled a base peak ion. Compounds are numbered according to *Rt* and then sorted according to chemical classes

#### Phenolic acids and derivatives

Many *Ipomoea* species are well recognized for their richness in phenolic acids [32]. In the present study, phenolic acids were classified into two distinct subgroups: a major group of hydroxycinnamic acid-containing compounds (29 metabolites) and a minor group of hydroxybenzoic acid derivatives (five metabolites).

#### Hydroxycinnamic acids and derivatives

Free hydroxycinnamic acids appeared as scattered nodes in the molecular network and were detected in most of the investigated species. These were tentatively annotated as coumaric acid isomers (**5** and **19**), caffeic acid isomers (6 and 33), dimethoxycinnamic acid (105), and ferulic acid (116). Their MS/MS spectra were characterized by common fragment ions at [M–H–18]⁻ and [M–H–44]⁻, corresponding to the neutral losses of H₂O and CO₂, respectively. Compounds 105 and 116 additionally exhibited a fragment ion at [M–H–14]⁻, attributed to the loss of a CH₂ moiety. Furthermore, compound 108 generated a diagnostic fragment ion at *m/z* 193 [ferulic acid–H]⁻ following the loss of 80 Da, indicative of sulfate elimination. Accordingly, this compound was tentatively identified as ferulic acid *O*-sulfate.

Like their aglycones, glycosylated hydroxycinnamic acid derivatives were also visualized as scattered nodes in the molecular network, likely due to their limited fragmentation patterns. These included caffeic acid-*O*-glucoside (14), dimethoxycinnamic acid di-*O*-hexoside isomers (21 and 30), dimethoxycinnamic acid tri-*O*-hexoside (60), and ferulic acid-*O*-trihexoside (103). All compounds exhibited successive neutral losses of hexose units (162n Da), yielding characteristic fragment ions at *m/z* 179, 207, and 193, corresponding to caffeic, dimethoxycinnamic, and ferulic acid moieties, respectively.

In addition, a diverse series of mono-, di-, and tri-substituted quinic acid esters were detected, consistently producing diagnostic fragment ions at *m/z* 191 [quinic acid–H]⁻ and *m/z* 173 [quinic acid–H–H₂O]⁻. They are widely distributed among the species studied, however, they are either absent or detected only in trace amounts in *I. carnea*, forming cluster W1 of the MNW and cluster C3 of the MNC (Fig. 4). Mono-substituted derivatives included coumaroyl (9; *m/z* 337.002), feruloyl (10; *m/z* 367.01), and caffeoyl (26; *m/z* 352.969) quinic acids, each displaying neutral losses corresponding to their respective dehydrated acyl moieties (146, 176, and 162 Da). They were reported before in *I. batatas* [6]. Similarly, di-substituted esters comprised dicaffeoyl isomers (39 and 66; *m/z* 514.93), caffeoyl coumaroyl (50; *m/z* 498.93), and caffeoyl sinapoyl (51; *m/z* 558.95) quinic acids. This kind of substitution was previously reported in *I. batatas*, *I. cairica*, and *I. pes-caprae* [6, 30, 64]. Meanwhile, tri-substituted derivatives were annotated as diferuloyl caffeoyl (31) and tricaffeoyl (64) quinic acids.

Furthermore, some hydroxycinnamic acid amides were identified, including *N*-feruloyl isomers (44 and 84), *N*-caffeoyl (73; *m/z* 312.25), *N*-coumaroyl (81), and *N*-sinapoyl (85) tyramines. These compounds were characterized by a neutral loss of dehydrated tyramine unit (119 Da). In contrast, compound 109 (*m/z* 293.11), tentatively assigned as *N*-sinapoylputrescine, exhibited a diagnostic loss of dehydrated putrescine (87 Da), further substantiating the presence of the fragmentation pattern of sinapic acid (*m/z* 223, 205, 195, 179, 177, 171). Compounds 44, 73, and 84 have been previously reported only in the sweet potato plant [6].

Finally, two aldehydes were tentatively identified, namely *p*-coumaraldehyde (99) and sinapaldehyde (101).

#### Hydroxybenzoic acids and derivatives

In parallel, hydroxybenzoic acid–related metabolites were annotated, mostly in all studied species, including dihydroxybenzaldehyde (8), hydroxybenzaldehyde (17), dihydroxybenzoic acid (22), and vanillic acid (43), all of which were reported recently in some *Ipomoea* species (Table 3) [69].

Notably, a complex hydroxybenzoic acid conjugate, dihydroxybenzoic acid-*O*-hydroxybenzoyl glucoside (62; *m/z* 434.98), was exclusively detected in *I. ochracea*. Its MS/MS spectrum exhibited sequential neutral losses consistent with glycosidic and benzoyl moieties, allowing its tentative annotation.

#### Flavonoids

In the current study, the investigated *Ipomoea* species exhibited remarkable flavonoid diversity, with a total of 68 annotated compounds. These comprised flavonoid *C*-glycosides, *O*-glycosides, and aglycones, with the notable and exclusive detection of sulfated flavonols and flavolignans in *I. ochracea*, highlighting its distinct metabolic signature.

#### Flavonoid C-glycosides

The flavone *C*-glycoside structures are well known in *Ipomoea* species, particularly in *I. aquatica* [26], *I. batatas* and *I. imperati* [27]. In the current study, they accumulated in all cultivated taxa and the wild *I. eriocarpa*, represented as clusters (W7 and C5) and self-looped nodes (W11 and C9) (Fig. 5). The mono*-C*-glucoside moieties include the 8-*C-*glucoside (12) and 6-*C-*glucoside (47) of luteolin methyl ether, orientin (25), isoorientin (29), as well as vitexin (40). In contrast, the di-*C*-glycosylated apigenin derivatives (16: apigenin 6-*C-*arabinose-8-*C*-glucose) and (20: apigenin 6-*C-*glucose-8-*C*-arabinose) were characterized for *I. eriocarpa* and *I. pes-caprae* (Table 3; Fig. 5). Moreover, flavanone-*C*-glycosides such as *C*-glucosides of eriodictyol (18), naringenin (24) were also detected. Furthermore, vitexin 7-*O*-glucoside (45) was recorded as the only *C*-glycosyl flavonoid-*O*-glycoside structure reported for the first time in *Ipomoea* species (Table 3).

#### Flavonoid-O-glycosides

Flavonoid-*O*-glycosides constituted the largest flavonoid subclass, encompassing flavone-, flavonol-, flavanone-, and acylated flavonoid glycosides. Flavone-*O-*glycoside derivatives of apigenin, luteolin, and chrysoeriol were commonly encountered, consistent with prior reports describing these compounds as dominant constituents of *Ipomoea* [29]. They are represented as clusters (W2 and C1) and some scattered self-looped nodes, such as C13 (Fig. 5). Based on MS/MS, GNPS libraries, and/or reference standards, they were tentatively identified as apigenin 7-*O*-rutinoside (68), apigenin 7-*O*-pentosyl glucoside (69), apigenin 7-*O*-glucoside (70), chrysoeriol-*O-*glucoside (76), chrysoeriol di-*O-*glucoside (111), and apigenin-*O*-pentoside (129). Likewise, flavonol-*O*-glycosides derived mainly from quercetin, kaempferol, and isorhamnetin were annotated, displaying diverse glycosylation patterns involving glucose, rhamnose, arabinose, xylose, sophorose, and rutinoside moieties. These compounds represented glycoside derivatives of quercetin (27, 32, 35, 37, 38, 42, 48, and 77), kaempferol (41, 46, 49, 53, 54, and 63), and isorhamnetin (58, 79, and 83). Lastly, trihydroxy trimethoxy flavone-*O*-glucoside (65) and monohydroxy hexamethoxy flavone-*O*-glucoside (72), as well as a flavanone *O*-glycoside, exemplified by eriodictyol 7-*O*-rutinoside (91), were less abundant but consistent with the first phytochemical findings in *Ipomoea* species.

#### Flavonol-O-acyl glycoside

Acylated flavonol glycoside structures were also detected, particularly characterized for *I. tricolor*. They represented as acetyl *O*-acetyl glucoside derivatives of kaempferol (52) and quercetin (59), *O*-malonyl glucoside of kaempferol (55), *O*-coumaroyl glucoside of kaempferol (90), *O*-caffeoyl glucoside of kaempferol (106), as well as *O*-feruloyl pentoside of quercetin (56).

#### Flavonoid aglycones

Flavonoid aglycones were represented in several subclasses, including flavones, flavonols, flavanones, dihydroflavonols, and polymethoxylated derivatives. Figure 6 shows significant clusters (W3, W4, W5, W9 & W10) of the MNW and (C4, C7, & C12) of the MNC. Common flavones such as apigenin (94) and luteolin (95), along with other methylated analogues, i.e. chrysoeriol (75) and tricin (80), were detected. Likewise, flavonols, including isorhamnetin (74), quercetin (78), kaempferol (102), and myricetin (92), were also abundant. Additionally, dihydroflavonols such as dihydromyricetin (57), taxifolin (23), and dihydrokaempferol (71), as well as a flavanone (naringenin 88), further expand the flavonoid core diversity and have been previously reported in selected *Ipomoea* taxa, albeit less frequently than their unsaturated counterparts [26].

Polymethoxylated flavones constituted a chemically distinctive subgroup, including di- to octa-methoxylated derivatives. Such compounds are known to enhance lipophilicity and membrane permeability and have been sporadically reported in *Ipomoea*, particularly in stress-adapted species [76]. Herein, they annotated as monohydroxy trimethoxy flavone (28), trimethoxy flavone (34), dihydroxy trimethoxy flavone (86), kaempferol dimethyl ether (89), quercetin dimethyl ether (96), luteolin dimethyl ether (115), and tetrahydroxy dimethoxy flavone (120) (Table 3).

Markedly, sulfated flavonol aglycones, ombuin-3-sulphate (82), kaempferol dimethyl ether-3-sulphate (97), and rhamnetin-3-*O*-sulphate (107), were exclusively detected in *I. batatas* and *I. cairica* (Table 3; Fig. 6).

#### Flavolignans

Flavolignans are generally absent from the genus *Ipomoea*, however, in this study, they were notably detected exclusively in *I. ochracea*, representing cluster (C8) of MNC (Fig. 5). Herein, they were confirmed by significant fragment ions of tricin at (*m/z* 329 and 299), after loss of 238, 196, and 210 Da, indicating the *O*-acetyl guaiacyl glycerol (87), *O*-guaiacyl glycerol (104), and *O*-dimethoxy phenyl glycerol (110) derivatives, respectively.

#### Data analysis

**Fig. 7:**
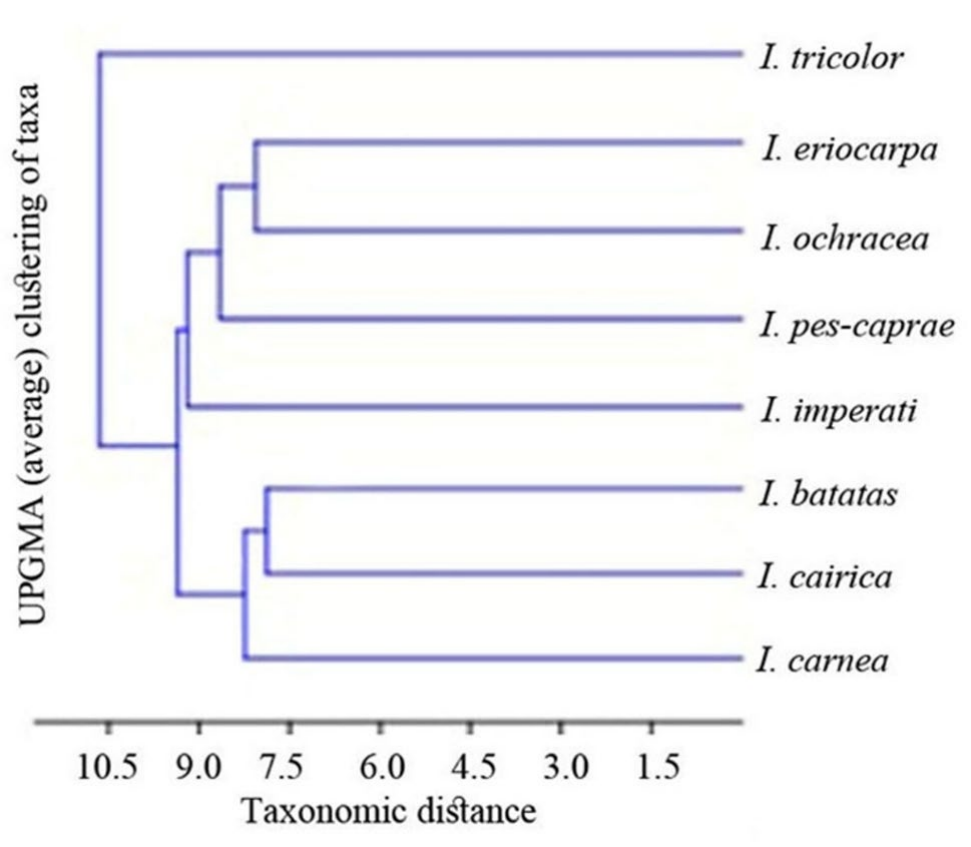
Dendrogram based on combined macro- and micromorphological as well as phytochemical characters of the studied species

The obtained dendrogram based on macro-, micromorphological and phytochemical characters of the studied species reveals a hierarchical structure with varying degrees of similarity among the eight studied species (Fig. 7). *Ipomoea tricolor* becomes clearly separated from all other species and connects the rest of the assemblage only at relatively high linkage distances. The remaining species form a large cluster, which is further divided into two major groups. The first group includes taxa *I. eriocarpa*,* I. ochracea*,* I. pes-carpae* and *I. imperati*, which display close affinities, while the second group includes *I. batatas*,* I. cairica*, and *I. carnea* grouped with relatively short linkage distances. The close relationship between *I. eriocarpa* and *I. ochracea*, within the first group, as well as *I. batatas* and *I. cairica*, in the second group, form tight subgroups, indicating strong similarity.

## Discussion

### Morphological interpretation

The genus *Ipomoea* exhibits a remarkable range of growth habits, varying from annual herbs and woody vines (lianas) to shrubs and small trees [51]. All the characters recorded in this study revealed significant variations among the eight studied species of *Ipomoea.* The vine habit in genus *Ipomoea* is considered the ancestral (plesiomorphic) state, and the shrubby habit is apomorphic [47, 80]. In the present study, *I. carnea* is considered the only species that exhibits a shrubby and erect growth habit (Table 2; Fig. 1; c_1_). Based on our direct observation and monitoring, *I. carnea* exhibits a twining habit under crowded conditions, the individuals of this species may exhibit this behavior as a shade avoidance and competition response, growing toward and around neighbors to access light and space. This shift in growth form reflects stem plasticity due to environmental cues and competition for resources. However, this habit is lost, and the stem becomes erect when the plant grows individually in open spaces, this observation agrees with the conclusions of McDonald [80]. Leaf composition of *I. batatas* and *I. cairica is* palmatifid and palmatipartite leaves, respectively (Table 2; Fig. 1; a_1_ & b_1_); our findings are in accordance with the observation of Mabberley [81]. The fleshy leaf was observed in *I. imperati* and *I. pes-caprae* (Table 2; Fig. 1; e_1_ & g_1_), which is regularly associated with environmental adaptation to high light intensity and water-limited habitats [9]. Nectar-producing glands at the base of the leaf lamina are recorded only in *I. pes-caprae* (Table 2), and the primary functions of these glands are ecological adaptation and indirect defense against herbivory [82, 83].

Regarding the micromorphological characters, the striated cuticle was found in the stems of *I. batatas*,* I. cairica*,* I. carnea* and *I. eriocarpa* (Table 2; Fig. 1; a_3_-d_3_), which is in accordance with Dos Santos et al. [84]. These striations are special architectural structures on the epidermal cells and have a vital protective function [85]. The recorded hypodermis in *I. batatas* and *I. cairica* (Table 2; Fig. 1; a_3_ & b_3_) has an important role in storage and structural support as reported previously by Fahn [86] and Dos Santos et al. [84]. Metcalfe and Chalk [9] noted that the simple and capitate glandular trichomes are recorded in the genus *Ipomoea*. Both types are recorded in the studied species except in *I. cairica* and *I. imperati*, where they are absent (Table 2; Fig. 1; a_2−4_-h_2−4_). Our results are consistent with previous studies by Folorunso [85] and Bolarinwa et al. [87], who considered trichomes a reliable diagnostic character for differentiating *Ipomoea* species alongside its vital role in environmental adaptation as barriers against sunlight and water loss [88]. The presence of ring-porous xylem vessel distribution is correlated with the vine habit, which was recorded in all studied species except for *I. carnea* (Table 2). These results are consistent with previous reports by Lowell and Lucansky [89], Angyalossy et al. [90], Martins et al. [91], but contradict those of Carlquist and Hanson [11], Rajput et al. [14]. Bicollateral vascular bundles were a characteristic feature in the genus *Ipomoea*, in general, and were observed in all species under study. They are marked by the presence of the inner phloem (intra-xylary phloem). This trait contributes to the efficiency of the nutrient translocations, which in turn enhances adaptation and facilitates the wider distribution of the genus *Ipomoea* [92, 93]. The schizogenous ducts are significant anatomical features in the pharmacognostical plant identification. They are abundant in the stem anatomy of all studied species except *I. cairica* and *I. eriocarpa*, where they occurred in low numbers (Table 2; Fig. 1, a_3_-h_3_). In contrast, they are absent in the lamina of *I. batatas*,* I. cairica* and *I. eriocarpa* (Table 2; Fig. 1, a_4_-h_4_). This finding agrees with Sahu and Kohli [94], who documented that schizogenous ducts are less frequent in the lamina than in the stem. The tanniniferous cells observed in *I. imperati* and *I. pes-caprae* (Table 2) serve as chemical defenses against herbivory and pathogens [84]. Calcium oxalate crystals, predominantly in the form of druses, were abundantly observed in all species studied (Table 2). They considered a functional structure rather than just a waste deposit, since they play important roles in plant defense against herbivores, as well as in stomatal closure and reduction of water loss [94–96].

The results reported an isolateral mesophyll in *I. imperati* and *I. pes-caprae* (Table 2), which notably possess succulent leaves compared to the other studied species. This anatomical feature may represent an adaptive strategy to regulate high solar radiation, as isolateral mesophyll enhances photosynthetic efficiency under intense light conditions. These findings are reliable with those reported by Silva et al. [24], who highlighted the ecological significance of such adaptations in plants inhabiting exposed environments. The rest of the species have dorsiventral mesophyll, which is consistent with Ekeke et al. [97] and Dos Santos et al. [84]. In general, there is a relation between the fleshy leaves and the isolateral mesophyll [9]. The studied species exhibit two types of stomata (paracytic and anisocytic); the heterostomatic nature is noted in *Ipomoea* species. Regarding the distribution of stomata (Figs. 2 and 3), all studied species are amphistomatic, which is a common character in the genus *Ipomoea* and the family in general [10, 98].

### Phytochemical investigation

*Ipomoea* species have been previously reported to contain a wide range of chemical structures, including phenolic acids and flavonoids. These compounds were mostly described in *I. batatas* [24, 25, 64, 99, 100–104], *I. carnea* [105], *I. cairica* [30, 106, 107] and *I. pes-caprae* [29, 69, 108, 109], but little chemical characterization was reported for *I. eriocarpa*,* I. imperati*, *and I. tricolor.* In contrast, chemical insights on *I. ochracea* are still lacking. In this study, LC-MS analysis, using the GNPS molecular networking tool, expanded the scope of phytochemical analysis of the studied species, revealing their metabolic characteristics and highlighting the extent of their chemical diversity, which is documented here for the first time. This analysis also presents the first comprehensive chemical characterization of *I. ochracea*.

The comprehensive profiling of phenolic acids revealed pronounced chemodiversity among the investigated *Ipomoea* species. As summarized in Table 3, hydroxycinnamic acid derivatives represented the dominant phenolic acids subclass, both in terms of structural diversity (29 metabolites) and distribution frequency across species. A clear distinction was observed between wild and cultivated *Ipomoea* species regarding phenolic composition and structural complexity. Cultivated species exhibited a higher abundance and broader diversity of hydroxycinnamic acid esters, especially mono-, di-, and tri-acylated quinic acid esters (e.g., compounds 26, 50, 64). These highly substituted derivatives have been reported before in *I. batatas*, suggesting an enhancement of the phenylpropanoid pathway [103]. However, the reduced presence or trace detection of complex quinic acid esters in some wild taxa, especially *I. carnea*, indicates interspecific metabolic specialization.

In contrast, several phenolics, such as free hydroxycinnamic acids (5, 6, 19, 33), dicaffeoyl quinic acid isomers (39, 66), N-feruloyltyramine isomers (44, 84), and ferulic acid-O-sulfate (108), were widely distributed and detected in both wild and cultivated species. They appeared more consistently in cultivated taxa, possibly reflecting adaptive or selective pressures linked to domestication, stress tolerance, or nutritional enhancement. Other derivatives exhibited species-specific or restricted occurrence patterns, particularly certain glycosylated forms (21, 30, 60, 103), reflecting metabolic specialization. Compared to hydroxycinnamic acids, hydroxybenzoic acid derivatives (five metabolites) were less diverse but broadly distributed, indicating their conserved yet minor contribution to the overall phenolic profile.

In general, cultivated species demonstrated greater phenolic structural elaboration, while wild species maintained a more conserved but less diversified phenolic pattern, highlighting the potential impact of domestication on secondary metabolite diversification.

Flavonoids represented the most structurally diverse and taxonomically informative class of secondary metabolites detected across the investigated *Ipomoea* species. At the genus level, flavone and flavonol aglycones and glycosides clearly predominated, confirming their central role in the phenolic profile of *Ipomoea* and supporting previous phytochemical reports that recognize these subclasses as characteristic constituents of the genus [101, 102, 104, 106–110].

A consistent pattern observed was the widespread occurrence of flavonoid *C*-glycosides in all cultivated taxa and in *I. eriocarpa*. The stability of the *C*–*C* glycosidic bond and the recurrent detection of these compounds suggest a conserved biosynthetic capacity within these groups, potentially reflecting shared metabolic regulation or phylogenetic proximity. In contrast, flavonoid *O*-glycosides, particularly quercetin- and kaempferol-based derivatives, displayed high structural diversity and broad distribution among species, representing a common metabolic hallmark of the genus.

Flavonoid aglycones exhibited moderate chemodiversity compared to hydroxycinnamic acid derivatives and flavonoid glycosides. Certain aglycones were detected across different species, indicating conserved flavonoid biosynthetic pathways within the genus. However, their relative abundance and co-occurrence with methylated derivatives differed between wild and cultivated taxa. Cultivated species exhibited a broader spectrum of flavonoid aglycones, particularly O-methylated derivatives (28, 34, 80, 115). These compounds represent advanced methylation steps in the flavonoid biosynthetic pathway, suggesting enhanced enzymatic modification and metabolic specialization in cultivated taxa [101]. In contrast, wild species were characterized predominantly by non-methylated flavonoid aglycones (23, 57, 71, 74, 78, 89). These compounds represent more conserved core flavonoid skeletons.

Species-specific trends further enhanced chemotaxonomic resolution. Acylated flavonol glycosides were especially prominent in *I. tricolor*, indicating a higher degree of flavonoid structural modification in this species. These kinds of compounds have been previously reported in *I. batatas* [102]. Sulfated flavonol aglycones were exclusively detected in *I. batatas* and *I. cairica*, suggesting species-specific sulfotransferase activity and highlighting their potential value as discriminative chemotaxonomic markers [111]. The isolation of these structures was confirmed previously in *I. cairica* [106]. Notably, tricin-derived flavolignans were found only in *I. ochracea*, revealing a distinct metabolic specialization likely linked to unique coupling between flavonoid and phenylpropanoid pathways. This restricted occurrence reinforces the metabolic individuality of *I. ochracea* and supports its separation from closely related species.

Overall, the observed phenolic and flavonoid distribution patterns underscore both the conserved metabolic framework of the genus and the presence of species-level chemical signatures with clear taxonomic relevance.

#### Data analysis interpretation

The resulting dendrogram (Fig. 7) reveals clear patterns of phenetic similarity among the eight species studied, forming a hierarchical structure with varying degrees of similarity. *Ipomoea tricolor* is clearly separated from all other species, joining the remaining assemblage only at relatively high linkage distances. This marked separation indicates that *I. tricolor* possesses a unique combination of morphological and phytochemical characters not shared with the other species. Anatomically, its lamina is characterized by both glandular and e-glandular trichomes with a multicellular base (Table 2). Phytochemically, it is the only species producing dihydroflavone-*C-*glycosides (compounds 18 and 24), flavonol-*O*-acyl glycosides (compounds 49, 52, 55, 56, 90, and 106), as well as some phenolic acid derivatives (compounds 101, 103, and 105) (Table 3). This distinctive metabolite profile highlights the taxonomic significance of specialized secondary metabolites as indicators of evolutionary divergence, likely associated with lineage-specific biosynthetic pathways involving *C*-glycosyltransferases and acyltransferases. These metabolites may confer adaptive advantages, such as enhanced stability and stress tolerance [112]. Collectively, these findings support the placement of *I. tricolor* within the defined section “Tricolores” of the subgenus “Quamoclit” [46], while also suggesting an independent metabolic route and a distinct evolutionary lineage within the genus [113].

The remaining species form a large cluster with relatively short linkage distances, supporting their placement within sub-genus “Eriospermum”, except *I. cairica* and *I. eriocarpa*, which are assigned to sub-genus “Quamoclit” [46]. This cluster is further divided into two major groups. The first group includes taxa *I. eriocarpa*,* I. ochracea*,* I. pes-carpae* and *I. imperati*, all belonging to section “Erpipomoea” of sub-genus “Eriospermum”, except for *I. eriocarpa* [46]. Similarly, the second group includes *I. batatas*,* I. cairica*, and *I. carnea*, largely corresponding to section “Eriospermum” of sub-genus “Eriospermum”, except for *I. cairica* [46]. Within the first group, the close relationship between *I. eriocarpa* and *I. ochracea* reflects a high degree of similarity in both morphological and chemical characters, including an acuminate lamina apex, e-glandular trichomes with a multicellular base in the lamina anatomy (Table 2), and the exclusive presence of apigenin (94) (Table 3). The smooth, hierarchical joining of *I. pes-carpae* and *I. imperati* to the tight pair (*I. eriocarpa* and *I. ochracea*) suggests continuous morphological and chemical variation. The elevated anticlinal walls, the depressed periclinal wall, and notched lamina apex distinctly separate *I. imperati*, supported by the occurrence status of some flavonol-*O*-glycosides, such as the presence of compound (32) and the absence of (48), as well as the clear detection of a sulphated coumarin (15). Whereas *I. pes-caprae* exhibits a marginate lamina apex, nectar-producing glands at the lamina base, and glandular trichomes in the stem anatomy. In contrast, in the second group, *I. batatas* and *I. cairica* form a tight subgroup. Both species share distinctive leaf forms, including palmatifid and palmatipartite leaves, respectively, as well as common anatomical features such as the presence of a hypodermis and the absence of schizogenous ducts. Additionally, druses are less abundant in these species compared to others. Their phytochemical similarity is further supported by the presence of sulphated flavonoids (82 & 107) and flavanone aglycone (88). The subsequent clustering of *I. carnea* suggests a degree of continuity in morphological and chemical characters, despite its distinguishing features, such as hollow pith cells, diffuse porous xylem vessel distribution, and the absence of certain phenolic derivatives (31).

The correspondence between morphological and metabolite data observed in the dendrogram underscores the strength of an integrative approach in species delimitation. By combining independent datasets, this method reduces uncertainty associated with single-data analyses and provides a more robust framework for interpreting taxonomic relationships. Notably, the placement of *I. eriocarpa* and *I. cairica* suggests that traditional classifications based solely on morphology may not fully reflect evolutionary relationships within the genus. Instead, the integration of phytochemical data offers additional resolution, indicating that these species may be more closely aligned with subgenus “Eriospermum” than previously assumed [45]. Finally, the characteristics documented here will aid future taxonomic revisions of the group, suggesting that the genus is not fully monophyletic.

## Conclusion

Diagnostic macro- and micromorphological characters alongside a diverse set of secondary metabolites (132 compounds) revealed substantial variation among the species, along with unique features for each one. This indicates that the genus *Ipomoea* shows a high degree of adaptability to different environmental conditions and its wide distribution. Such variability represents one of the main factors that has led to disagreement in its classification across taxonomic studies, which may also be related to its monophyletic nature. Dendrogram analysis revealed clear clustering patterns, with *I. tricolor* distinctly separated, supporting its unique taxonomic position, while the remaining species formed structured groups that generally conform to, and even improve upon, traditional classifications.

Overall, this study underscores the significance of combining morphological and metabolomic data in plant systematics, providing enhanced resolution and reliability in species delimitation. Future research incorporating broader species sampling, molecular phylogenetic markers, and biological assessments is recommended to validate a more comprehensive understanding of evolutionary relationships within *Ipomoea*.

## Supplementary Information

Supplementary Material 1.

## Data Availability

The voucher specimens of the collected plant species were deposited in CAIA and CAIRC herbaria, and their accession numbers are listed in Table (1). The resulting networks can be accessed through the links: https://gnps.ucsd.edu/ProteoSAFe/status.jsp?task=d4fb5c42dc3d43ee84ffee1d20c7d8ac, representing the molecular network of the four wild species (MNW), and https://gnps.ucsd.edu/ProteoSAFe/status.jsp?task=f22b87860e294468ab3eeb25e17de684, representing the molecular network of the four cultivated species. Furthermore, all generated and/or analyzed datasets `for the anatomical study, are included in the main text of the manuscript, while the datasets related to the phytochemical study are provided in the supplementary file.
