# Supplementary material for: Integrating morphological and phytochemical characters in understanding the taxonomic relationship within some Ipomoea L. species
Source: BMC Plant Biol. 2026 May 5;26:811. doi: 10.1186/s12870-026-08857-4 (PMC13147700; doi:10.1186/s12870-026-08857-4)
Supplement: Supplementary file 1 — Supplementary Material 1. [file 12870_2026_8857_MOESM1_ESM.docx]

**Supplementary data**

**Integrating morphological and phytochemical characters in understanding the taxonomic relationship within some *Ipomoea* L. species**

Mohamed A. Salim^1*^, Mona O. El Shabrawy ^2^, Salma S. Abd El-Ghany^1^, Mariam I. Hussein^1^, Mona M. Marzouk ^2^

^1^ Department of Botany, Faculty of Science, Ain Shams University, Cairo, Egypt

^2^ Department of Phytochemistry and Plant Systematics, National Research Centre, 33 El Bohouth St., P.O. 12622, Cairo, Egypt.

**Corresponding author:** Mohamed A. Salim

**e-mail:** [Mohamed.salim@sci.asu.edu.eg](mailto:Mohamed.salim@sci.asu.edu.eg)**, Orcid no.** 0000-0003-0967-5465**, Mobile:** +2-01008820375

**Table of contents**

| **Material and methods** | | 2 |
| --- | --- | --- |
| LC-ESI-MS/MS analysis, data processing, and metabolites annotation | | 2 |
| Molecular network creation and data visualization | | 2-4 |
| **Results** | | 4 |
| Metabolites annotation of other secondary metabolites and primary metabolites | | 4-5 |
| **Supplementary Figures** | |  |
| Figure S1 | Base peak chromatograms of the studied species: **a**; *Ipomoea batatas*, **b**; *I. cairica*, **c**; *I. carnea*, **d**; *I. eriocarpa*, **e**; *I. imperati*, **f**; *I. ochracea*, **g**; *I. pes-caprae*, **h**; *I. tricolor*. | 6 |
| Figure S2 | The molecular network of the wild *Ipomoea* species (MNW). | 7 |
| Figure S3 | The molecular network of the cultivated *Ipomoea* species (MNC). | 8 |
| **Supplementary Tables** | |  |
| Table S1 | Phytochemical constituents in *Ipomoea* species extract, using LC-MS/MS analysis, in a negative ionization mode and data matrix of the studied species. | 9-11 |
| **References** |  | 11-12 |

**Material and methods**

LC-ESI-MS/MS analysis, data processing, and metabolites annotation

The phytochemical constituents of the eight aqueous ethanol extracts of Ipomoea species were analyzed by LC-ESI-MS/MS (liquid chromatography-electrospray ionization–tandem mass spectrometry), through an Exion LC AC system for separation and a SCIEX Triple Quad 5500+ MS/MS system equipped with electrospray ionization for detection. The investigation was performed using a chromatographic column (C-18, 2.1 × 150 mm, 2.7 µm, Ascentis® Express 90 Å) (40 °C), applying an injection volume of 5 µl, with a flow rate set at 0.3 ml/min. The mobile phases consisted of solvent A (5 mM ammonium formate adjusted to pH 8) and solvent B (acetonitrile of HPLC grade). The chromatographic gradient was planned as follows: 0-1 min at 5% B, 1-20 min gradually increasing from 5% to 100% B, 20.01-25 min at 100% B, and 25.01-30 min returning to 5% A. MS/MS analysis was carried out in negative ion mode over a mass range of 100 to 1000 Da, using EMS-IDA-EPI for MS1 with parameters including -4500 V ion spray voltage, 25 psi curtain gas, source temperature at 500 °C, and 45 psi for ion source gases 1 and 2. For MS2, the mass range was set from 50 to 1000 Da, with a declustering potential of -80, a collision energy spread of 15, and a collision energy of -35. Peak detection and spectrum interpretation were performed with PeakView® 1.2 Software (SCIEX, Framingham, MA, USA). Compound identification was done manually by comparing MS data and retention times with literature data, alongside comparisons with GNPS libraries (Table 2) [1].

Molecular network creation and data visualization

Two negative molecular networks (Figures S1 and S2) were created using the online workflow (<https://ccms-ucsd.github.io/GNPSDocumentation/>) on the GNPS website (<http://gnps.ucsd.edu>). Raw data were converted into open-source files (.mzML format) using the MSConvert tool (ProteoWizard Software Foundation, Version 3.0.1933, USA) and subsequently uploaded to GNPS via WinSCP. Due to GNPS platform limitations, a single classical molecular networking job can include a maximum of six sets (G1-G6). As our dataset comprised eight samples plus one blank, which exceeded this limit, the analysis was split. The (.mzML) datasets of the four wild species (*I. cairica, I. carnea, I. eriocarpa, and I. imperati*) were grouped with the blank file in one network job, while those of the four cultivated species (*I. batatas, I. ochracea, I. pes-caprae,* and *I. tricolor*) were analyzed with the blank in a separate network job. This approach allowed accurate network construction, appropriate blank subtraction, and reliable comparison within each ecological group. For each job, the precursor ion mass tolerance was set to 2.0 Da, and a MS/MS fragment ion tolerance of 0.5 Da. Each network was then created*,* where edges were filtered to have a cosine score above 0.65 and more than 4 matched peaks. Further, the maximum size of a molecular family was set to 40. Finally, the MS^2^ spectra in the created networks were then searched against GNPS' spectral libraries, including reference spectra (GNPS-Collections), community spectral libraries generated (GNPS-community), and other third-party libraries (*i.e.* MassBank, Respect, and NIST) [2]. All matches kept between MS spectra and library spectra were required to have a score above 0.7 and at least 6 matched peaks. The resulting networks can be accessed through the links: [https://gnps.ucsd.edu/ProteoSAFe/status.jsp?task=d4fb5c42dc3d43ee84ffee1d20c7d8ac,](https://gnps.ucsd.edu/ProteoSAFe/status.jsp?task=d4fb5c42dc3d43ee84ffee1d20c7d8ac) representing the molecular network of the four wild species (MNW), and <https://gnps.ucsd.edu/ProteoSAFe/status.jsp?task=f22b87860e294468ab3eeb25e17de684>, representing the molecular network of the four cultivated species (MNC). In this approach, each node represents a spectrum (metabolite), while edges connect metabolites with similar spectral features, reflecting structural or chemical relationships. Details about the corresponding compound, which could be identified through GNPS libraries such as the library ID and the number of matching fragment ions were observed [2]. Visualization of the spectral network was then performed using Cytoscape 3.9.1, where each spectrum is represented as a node and the connections between nodes as edges, reflecting the structural relationships through the MS analysis. Within one node, each color corresponds to a single extract, and the ​​colors' areas reflect the relative abundance or intensity of a specific metabolite in all studied extracts. In addition to metabolite dereplication through the GNPS platform, the annotation process was further validated through multiple complementary approaches. Where possible, compounds were confirmed by comparison with authentic reference standards (Table 2). For putatively identified metabolites, fragmentation patterns were cross-referenced with published literature (Table 2) and searched against selected natural product databases, including ChemSpider (<http://www.chemspider.com/>) and PubChem (<https://pubchem.ncbi.nlm.nih.gov/>).

**Results**

**Metabolites annotation of other secondary metabolites and primary metabolites**

Coumarins and anthocyanidins were detected as a minor yet structurally informative class of secondary metabolites within the investigated *Ipomoea* species. Scopoletin-*O*-sulfate (**15**; *m/z* 270.92) was tentatively identified based on its characteristic fragmentation pattern, which involved the neutral loss of 80 Da corresponding to sulfate cleavage, yielding a diagnostic product ion at *m/z* 191 [M–H–80]⁻, consistent with the deprotonated scopoletin aglycone. In addition, scopoletin (**15**, *m/z* 270.92), trihydroxy coumarin (**93**, *m/z* 193.08), and dihydroxy coumarin (**98**, *m/z* 176.95) were also annotated. Coumarins have been previously reported in several *Ipomoea* species, where they are implicated in allelopathic interactions, antioxidant defense, and antimicrobial activity [3]. On the other hand, anthocyanidins were represented by cyanidin 3-*O*-hexose (**61**; *m/z* 449.98), which was exclusively annotated in *I. tricolor* based on its diagnostic neutral loss of 162 Da corresponding to hexose, yielding the aglycone cyanidin fragments. Anthocyanins are well-known contributors to pigmentation and photoprotection in *Ipomoea*, particularly in ornamental and stress-exposed species, and their presence supports earlier phytochemical reports on flower and leaf tissues of the genus [4].

In addition to all previously discussed secondary metabolites, a diverse set of amino, organic, and fatty acids was detected, reflecting core primary metabolism. Identified amino acids included arginine (1; *m/z* 173.06), valine (3; *m/z* 116.08), asparagine (4; *m/z* 131.01), leucine/isoleucine (7; *m/z* 130.03), and tryptophan (11; *m/z* 203.03), which were reported before in *Ipomoea* species [5]. These metabolites play fundamental roles in nitrogen metabolism, energy production, and the biosynthesis of secondary metabolites. Citric acid (**2**; *m/z* 191.01) was observed as the only detected organic acid and has been detected before in *I. batatas* [6]. Likewise, respective fatty acids and derivatives were detected, reflecting active lipid metabolism in the studied species. These included trihydroxy-octadecadienoic acid (**67**; *m/z* 327.13), oxo-octadecatrienoic acid (**112**; *m/z* 291.09), and hydroxyoctadecatrienoic acid (**117**; *m/z* 293.12). In addition, complex glycolipid-like structures were annotated, such as hydroxy-octadecatrienoyl-dihexosyl glycerol (**113**; *m/z* 691.06) and hydroxy-octadecatrienoic acid-*O*-dihexosyl conjugate (**114**; *m/z* 617.08). Fatty acids and derivatives have been previously reported in *Ipomoea* species, particularly under biotic or abiotic stress conditions [3]. Further lipid-type metabolites are phospholipid structures as hexadecanoyl-glycero-phospho-inositol (121; *m/z* 571.08) and linolenoyl-palmitoyl-phosphatidylglycerol (132; *m/z* 743.19). These membrane lipids are essential structural components of cellular and chloroplast membranes and play key roles in signal transduction and stress responses. Their occurrence supports earlier lipidomic studies reporting complex glycerophospholipids in *Ipomoea* leaves and aerial tissues [7]. The detection of these primary metabolites is consistent with previous metabolomic investigations of *Ipomoea* species and confirms the metabolic integrity of the analyzed samples [5-7].

*
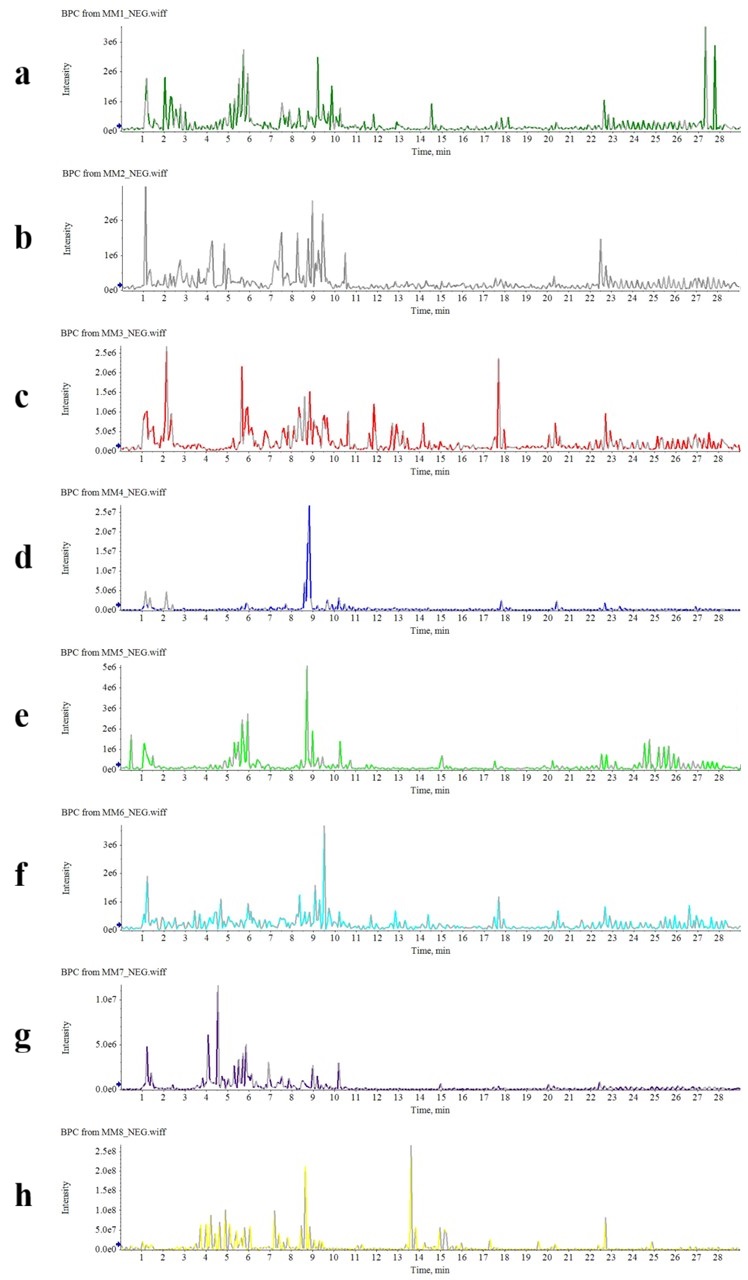
*

**Figure S1.** Base peak chromatograms of the studied species: **a**; *Ipomoea batatas*, **b**; *I. cairica*, **c**; *I. carnea*, **d**; *I. eriocarpa*, **e**; *I. imperati*, **f**; *I. ochracea*, **g**; *I. pes-caprae*, **h**; *I. tricolor*.

*
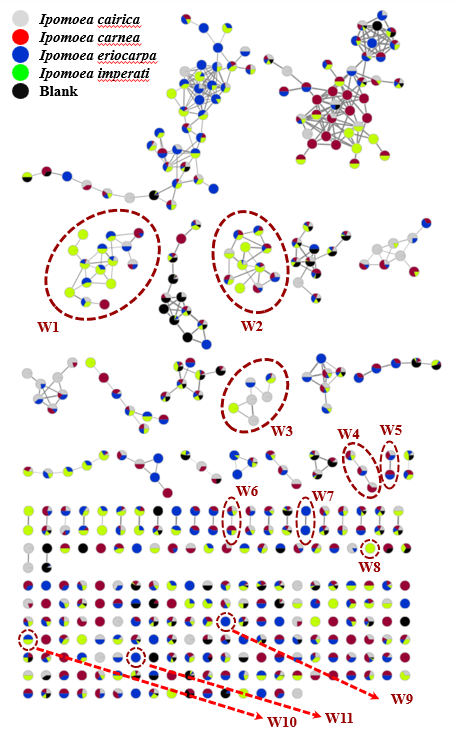
*

**Figure S2.** The molecular network of the wild *Ipomoea* species (MNW).

*
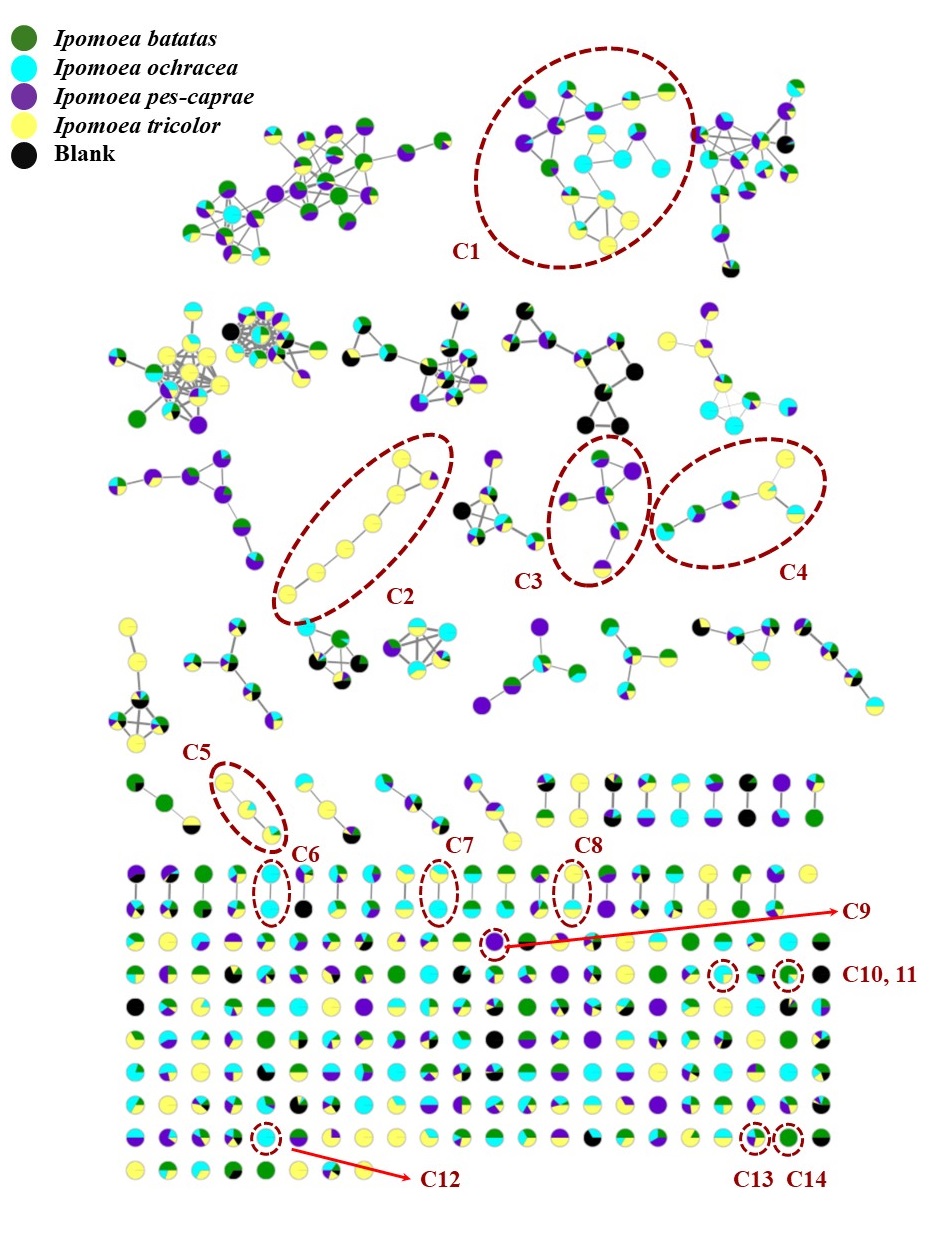
*

**Figure S3.** The molecular network of the cultivated *Ipomoea* species (MNC).

**Table S1.** Phytochemical constituents in *Ipomoea* species extract, using LC-MS/MS analysis, in a negative ionization mode and data matrix of the studied species.

| **Taxa**  **Chemical characters** | ***a*** | ***b*** | ***c*** | ***d*** | ***e*** | ***f*** | ***g*** | ***h*** |
| --- | --- | --- | --- | --- | --- | --- | --- | --- |
|  | 1 | 1 | 1 | 1 | 1 | 1 | 1 | 1 |
|  | 1 | 1 | 1 | 1 | 1 | 1 | 1 | 1 |
|  | 1 | 1 | 1 | 1 | 1 | 1 | 1 | 1 |
|  | 0 | 1 | 0 | 1 | 0 | 0 | 0 | 0 |
|  | 1 | 1 | 1 | 1 | 1 | 1 | 1 | 1 |
|  | 1 | 1 | 1 | 1 | 1 | 1 | 1 | 1 |
|  | 1 | 1 | 1 | 1 | 1 | 1 | 1 | 1 |
|  | 1 | 1 | 1 | 1 | 1 | 1 | 1 | 1 |
|  | 0 | 1 | 0 | 1 | 1 | 1 | 1 | 0 |
|  | 0 | 0 | 0 | 1 | 1 | 0 | 0 | 0 |
|  | 1 | 1 | 1 | 1 | 1 | 1 | 1 | 1 |
|  | 1 | 0 | 0 | 0 | 1 | 1 | 1 | 1 |
|  | 1 | 1 | 1 | 1 | 1 | 1 | 1 | 1 |
|  | 1 | 0 | 0 | 0 | 0 | 0 | 1 | 1 |
|  | 0 | 0 | 0 | 0 | 1 | 0 | 0 | 0 |
|  | 0 | 0 | 0 | 1 | 0 | 0 | 1 | 0 |
|  | 1 | 1 | 1 | 1 | 1 | 1 | 1 | 1 |
|  | 0 | 0 | 0 | 0 | 0 | 0 | 0 | 1 |
|  | 1 | 1 | 1 | 1 | 1 | 1 | 1 | 1 |
|  | 0 | 0 | 0 | 1 | 0 | 0 | 1 | 0 |
|  | 0 | 0 | 0 | 0 | 1 | 0 | 0 | 0 |
|  | 1 | 1 | 0 | 1 | 1 | 1 | 0 | 1 |
|  | 0 | 1 | 1 | 1 | 0 | 1 | 0 | 0 |
|  | 0 | 0 | 0 | 0 | 0 | 0 | 0 | 1 |
|  | 0 | 0 | 0 | 1 | 0 | 1 | 0 | 1 |
|  | 1 | 0 | 0 | 0 | 1 | 0 | 1 | 1 |
|  | 1 | 0 | 0 | 0 | 1 | 0 | 1 | 0 |
|  | 0 | 0 | 0 | 0 | 0 | 1 | 0 | 1 |
|  | 0 | 0 | 0 | 1 | 0 | 1 | 0 | 1 |
|  | 0 | 0 | 0 | 0 | 1 | 0 | 0 | 0 |
|  | 1 | 1 | 0 | 1 | 1 | 1 | 1 | 1 |
|  | 0 | 0 | 0 | 0 | 1 | 0 | 0 | 0 |
|  | 1 | 1 | 1 | 1 | 1 | 0 | 1 | 1 |
|  | 0 | 0 | 0 | 0 | 0 | 1 | 0 | 1 |
|  | 1 | 1 | 1 | 1 | 1 | 1 | 1 | 1 |
|  | 1 | 1 | 1 | 1 | 1 | 1 | 0 | 0 |
|  | 1 | 1 | 1 | 1 | 1 | 1 | 1 | 1 |
|  | 1 | 1 | 1 | 0 | 1 | 1 | 0 | 1 |
|  | 1 | 1 | 1 | 1 | 1 | 1 | 1 | 1 |
|  | 1 | 0 | 0 | 1 | 0 | 1 | 0 | 1 |
|  | 0 | 0 | 0 | 0 | 0 | 0 | 0 | 1 |
|  | 1 | 0 | 0 | 0 | 0 | 1 | 1 | 0 |
|  | 1 | 1 | 1 | 1 | 1 | 0 | 1 | 1 |
|  | 1 | 1 | 1 | 1 | 1 | 1 | 1 | 1 |
|  | 1 | 0 | 0 | 1 | 0 | 1 | 0 | 1 |
|  | 0 | 1 | 0 | 0 | 1 | 1 | 0 | 1 |
|  | 0 | 0 | 0 | 0 | 0 | 0 | 0 | 1 |
|  | 1 | 1 | 1 | 1 | 0 | 1 | 1 | 1 |
|  | 0 | 0 | 0 | 0 | 0 | 0 | 0 | 1 |
|  | 1 | 0 | 0 | 0 | 1 | 1 | 1 | 0 |
|  | 1 | 0 | 0 | 1 | 1 | 0 | 1 | 1 |
|  | 0 | 0 | 0 | 0 | 0 | 0 | 0 | 1 |
|  | 0 | 1 | 1 | 1 | 1 | 1 | 0 | 1 |
|  | 1 | 1 | 1 | 1 | 1 | 1 | 1 | 1 |
|  | 0 | 0 | 0 | 0 | 0 | 0 | 0 | 1 |
|  | 0 | 0 | 0 | 0 | 0 | 0 | 0 | 1 |
|  | 0 | 1 | 0 | 0 | 0 | 0 | 0 | 0 |
|  | 1 | 0 | 1 | 1 | 1 | 1 | 1 | 0 |
|  | 0 | 1 | 1 | 1 | 1 | 1 | 1 | 0 |
|  | 1 | 0 | 0 | 0 | 1 | 0 | 0 | 0 |
|  | 0 | 0 | 0 | 0 | 1 | 0 | 0 | 1 |
|  | 0 | 0 | 0 | 0 | 0 | 1 | 0 | 0 |
|  | 0 | 0 | 0 | 0 | 0 | 0 | 0 | 1 |
|  | 1 | 0 | 0 | 1 | 1 | 1 | 1 | 0 |
|  | 1 | 0 | 0 | 0 | 0 | 1 | 0 | 1 |
|  | 1 | 1 | 1 | 1 | 1 | 1 | 0 | 1 |
|  | 1 | 1 | 1 | 1 | 1 | 1 | 1 | 1 |
|  | 0 | 0 | 0 | 0 | 0 | 0 | 0 | 1 |
|  | 0 | 0 | 0 | 0 | 0 | 1 | 0 | 1 |
|  | 1 | 1 | 0 | 1 | 1 | 1 | 1 | 1 |
|  | 0 | 1 | 1 | 1 | 0 | 1 | 0 | 0 |
|  | 1 | 1 | 0 | 0 | 0 | 0 | 0 | 0 |
|  | 1 | 1 | 0 | 1 | 0 | 0 | 1 | 0 |
|  | 1 | 0 | 1 | 1 | 0 | 0 | 0 | 0 |
|  | 1 | 1 | 1 | 1 | 1 | 1 | 1 | 1 |
|  | 1 | 0 | 0 | 0 | 0 | 1 | 1 | 1 |
|  | 0 | 0 | 0 | 0 | 0 | 0 | 0 | 1 |
|  | 0 | 0 | 0 | 1 | 1 | 0 | 0 | 0 |
|  | 0 | 0 | 0 | 0 | 1 | 0 | 1 | 0 |
|  | 1 | 0 | 0 | 0 | 0 | 1 | 1 | 0 |
|  | 1 | 1 | 0 | 1 | 1 | 1 | 1 | 1 |
|  | 1 | 1 | 0 | 0 | 0 | 0 | 0 | 0 |
|  | 0 | 0 | 1 | 1 | 1 | 1 | 0 | 0 |
|  | 1 | 1 | 1 | 1 | 1 | 1 | 1 | 1 |
|  | 0 | 0 | 0 | 1 | 1 | 0 | 0 | 0 |
|  | 1 | 1 | 1 | 0 | 0 | 0 | 0 | 0 |
|  | 0 | 0 | 0 | 0 | 0 | 1 | 0 | 0 |
|  | 1 | 1 | 0 | 0 | 0 | 0 | 0 | 0 |
|  | 0 | 1 | 0 | 0 | 0 | 0 | 0 | 0 |
|  | 0 | 0 | 0 | 0 | 0 | 0 | 0 | 1 |
|  | 0 | 0 | 0 | 1 | 1 | 0 | 0 | 0 |
|  | 1 | 1 | 0 | 1 | 0 | 1 | 0 | 0 |
|  | 0 | 1 | 1 | 1 | 1 | 0 | 0 | 0 |
|  | 0 | 0 | 0 | 1 | 0 | 1 | 0 | 0 |
|  | 0 | 0 | 0 | 1 | 1 | 1 | 0 | 1 |
|  | 0 | 1 | 0 | 1 | 0 | 0 | 0 | 0 |
|  | 0 | 1 | 0 | 0 | 0 | 0 | 0 | 0 |
|  | 1 | 0 | 0 | 0 | 0 | 0 | 1 | 1 |
|  | 1 | 0 | 0 | 0 | 0 | 1 | 1 | 1 |
|  | 1 | 1 | 1 | 1 | 1 | 1 | 1 | 1 |
|  | 0 | 0 | 0 | 0 | 0 | 0 | 0 | 1 |
|  | 1 | 0 | 1 | 0 | 0 | 0 | 0 | 1 |
|  | 0 | 0 | 0 | 0 | 0 | 0 | 0 | 1 |
|  | 0 | 0 | 0 | 0 | 0 | 1 | 0 | 0 |
|  | 0 | 0 | 0 | 0 | 0 | 0 | 0 | 1 |
|  | 0 | 0 | 0 | 0 | 0 | 0 | 0 | 1 |
|  | 1 | 1 | 0 | 0 | 0 | 0 | 0 | 0 |
|  | 1 | 1 | 1 | 1 | 1 | 1 | 1 | 1 |
|  | 0 | 0 | 0 | 1 | 0 | 0 | 0 | 1 |
|  | 0 | 0 | 0 | 0 | 0 | 1 | 0 | 0 |
|  | 1 | 1 | 1 | 1 | 1 | 1 | 1 | 1 |
|  | 1 | 1 | 1 | 1 | 1 | 1 | 1 | 1 |
|  | 0 | 0 | 0 | 0 | 0 | 0 | 0 | 1 |
|  | 0 | 0 | 0 | 0 | 0 | 0 | 0 | 1 |
|  | 1 | 1 | 0 | 0 | 0 | 1 | 0 | 0 |
|  | 1 | 0 | 0 | 0 | 0 | 0 | 1 | 0 |
|  | 1 | 1 | 1 | 1 | 1 | 1 | 1 | 1 |
|  | 1 | 1 | 1 | 1 | 1 | 1 | 1 | 1 |
|  | 1 | 1 | 1 | 1 | 1 | 1 | 1 | 1 |
|  | 1 | 1 | 1 | 1 | 0 | 1 | 0 | 0 |
|  | 0 | 0 | 0 | 0 | 1 | 0 | 1 | 0 |
|  | 1 | 1 | 1 | 1 | 1 | 1 | 1 | 1 |
|  | 0 | 0 | 0 | 0 | 0 | 0 | 0 | 1 |
|  | 1 | 0 | 0 | 0 | 0 | 0 | 0 | 1 |
|  | 0 | 0 | 0 | 1 | 0 | 0 | 1 | 1 |
|  | 0 | 0 | 0 | 0 | 0 | 0 | 0 | 1 |
|  | 0 | 0 | 0 | 0 | 0 | 0 | 0 | 1 |
|  | 1 | 1 | 1 | 1 | 1 | 1 | 1 | 1 |
|  | 1 | 0 | 0 | 0 | 0 | 1 | 0 | 1 |
|  | 1 | 1 | 1 | 1 | 1 | 1 | 1 | 1 |
|  | 1 | 1 | 1 | 1 | 1 | 1 | 1 | 1 |
|  | 0 | 0 | 0 | 0 | 0 | 0 | 0 | 1 |
| **a:** *Ipomoea batatas*, **b:** *I. cairica*, **c:** *I. carnea*, **d:** *I. eriocarpa*, **e:** *I. imperati*, **f:** *I. ochracea*, **g:** *I. pes-caprae*, **h:** *I. tricolor*. **(0)** absent, **(1)** present | | | | | | | | |

**References**

1. Ragab NA, Ibrahim FM, El Shabrawy MO, Mahomoodally MF, Marzouk MM. Biotechnological application of African mustard (*Brassica tournefortii*): LC-ESI-MS/MS in synergism with featured-based molecular networking, antioxidant and anti-inflammatory propensities. *Biocatalysis and Agricultural Biotechnology*, 2025, 65, p.103533.
2. Wang M, Carver JJ, Phelan VV, Sanchez LM, Garg N, Peng Y, Nguyen DD, Watrous J, Kapono CA, Luzzatto-Knaan T, Porto C. Sharing and community curation of mass spectrometry data with Global Natural Products Social Molecular Networking. *Nature Biotechnology*, 2016, 34(8), pp.828-837.
3. Sulaiman CT, Deepak M, Sunil AR, Lijini KR, Balachandran I. Characterization of coumarins from *Ipomoea mauritiana* Jacq by LC-APCI-MS/MS analysis and evaluation of its anti-amnesic activity. *Beni-Suef University Journal of Basic and Applied Sciences*. 2019, 8. 10.1186/s43088-019-0022-z.
4. Zehra A, Zhou J, Ma H, Liu B, Sahito ZA, Wang Y, Yang W, Zhang L. Unraveling anthocyanin accumulation in sweet potatoes with integrated omics. *Scientia Horticulturae*, 2025, 350, p.114293.
5. Khatiwora E, Adsul VB, Deshpande NR. Identification of amino acids present in the leaves and stem of *Ipomoea carnea*. *Asian Journal of Chemistry,* 2009, 21 (6), 4409-4412.
6. Galdón BR, Ríos Mesa D, Rodríguez Rodríguez EM, Díaz Romero C. Influence of the cultivar on the organic acid and sugar composition of potatoes. *Journal of the Science of Food and Agriculture*, 2010, 90 (13), pp.2301-2309.
7. Li Z, Zhang R, Jiang X, Liu Y., Wang Z. Lipidomic Profiling of Sweet potato During Different Developmental Stages Using LC-ESI-MS/MS. *Foods*, 2025, 14(23), p.4109.
